# Supplementary material for: One-Pot Synthesis and Evaluation of Antioxidative Stress and Anticancer Properties of an Active Chromone Derivative
Source: Molecules. 2023 Mar 31;28(7):3129. doi: 10.3390/molecules28073129 (PMC10096453; doi:10.3390/molecules28073129)
Supplement: Supplementary file 1 [file molecules-28-03129-s001.zip › molecules-2290780-supplementary.pdf]

One-Pot Synthesis and Evaluation of Antioxidative Stress and Anticancer Properties of an Active Chromone Derivative

Chirattikan Maicheen[1], Chokchaloemwat Churnthammakarn[2], Nichapat Pongsroypech[2], Thitiphong Khamkhenshorngphanuch[2,3], Jiraporn Ungwitayatorn[4], Kanin Rangsangthong[2], Rathapon Asasutjarit[2,3], Sewan Theeramunkong[*2,3]

1 *Department of Pharmaceutical Chemistry, Faculty of Pharmaceutical Sciences, Huachiew Chalermprakiet University, Samut Prakarn 10540, Thailand*

2 *Faculty of Pharmacy, Thammasat University, Pathumthani, 12120, Thailand*

3 *Thammasat University Research Unit in Drug, Health Product Development and Application (DHP-DA), Department of Pharmaceutical Sciences, Faculty of Pharmacy, Thammasat University, Pathumthani, 12120, Thailand.*

4 *Department of Pharmaceutical Chemistry, Faculty of Pharmacy, Mahidol University, Bangkok 10400, Thailand*

*Corresponding author, e-mail: sewan@tu.ac.th

Received [3 March 2023]

Table of Contents

		Page
1.	General material and method	S3
2.	^{1}H-NMR spectrum of synthetic compounds	S4-S15
3.	^{13}C-NMR spectrum of synthetic compounds	S16-S27
4.	LCMS spectrum of synthetic compounds	S28-S32

1. General Materials and Methods

All reactions, unless otherwise indicated, were carried out in single-neck, round-bottom flasks fitted with a rubber septum and equipped with a magnetic stir bar. Air or water-sensitive solvents were transferred via a syringe. When required, solvents were degassed by bubbling nitrogen through a needle. A rotary evaporator concentrated the resulting organic layers at 45-70 °C under reduced pressure. The progress of the reaction was monitored with thin-layer chromatography (TLC) using a silica gel 60 F254 aluminum sheet. Visualization was performed with a 254 / 365 nm UV light source (Camag, Switzerland), generally followed by immersion in potassium permanganate ($KMnO_4$) or iodine vapor, as appropriate. Upon completion of the reaction, the crude products were prepared with a suitable procedure and finally dried and purified by column chromatography to provide a pure product.

The FTIR spectrum of the compounds was obtained by the KBr technique using an FTIR spectrometer (IRTracer-100 Shimadzu, Japan). The NMR spectrum was performed using an Ascend TM600/Advance III HD, Bruker, Switzerland. The spectrum of compounds was taken from the 4000 cm^{-1} to 650 cm^{-1} wave number range. ESI-TOF-MS was performed using a time-of-flight mass spectrometer (micrOTOF-Q-II, BrukerDaltonics, Germany). The ESI system positive-ion mode generated 200-800 m/z. The optimized mass spectrometric conditions included a gas temperature of 350 °C and a drying gas flow rate of 10.0 L/min.

2. ^{1}H-NMR spectrum of synthetic compounds

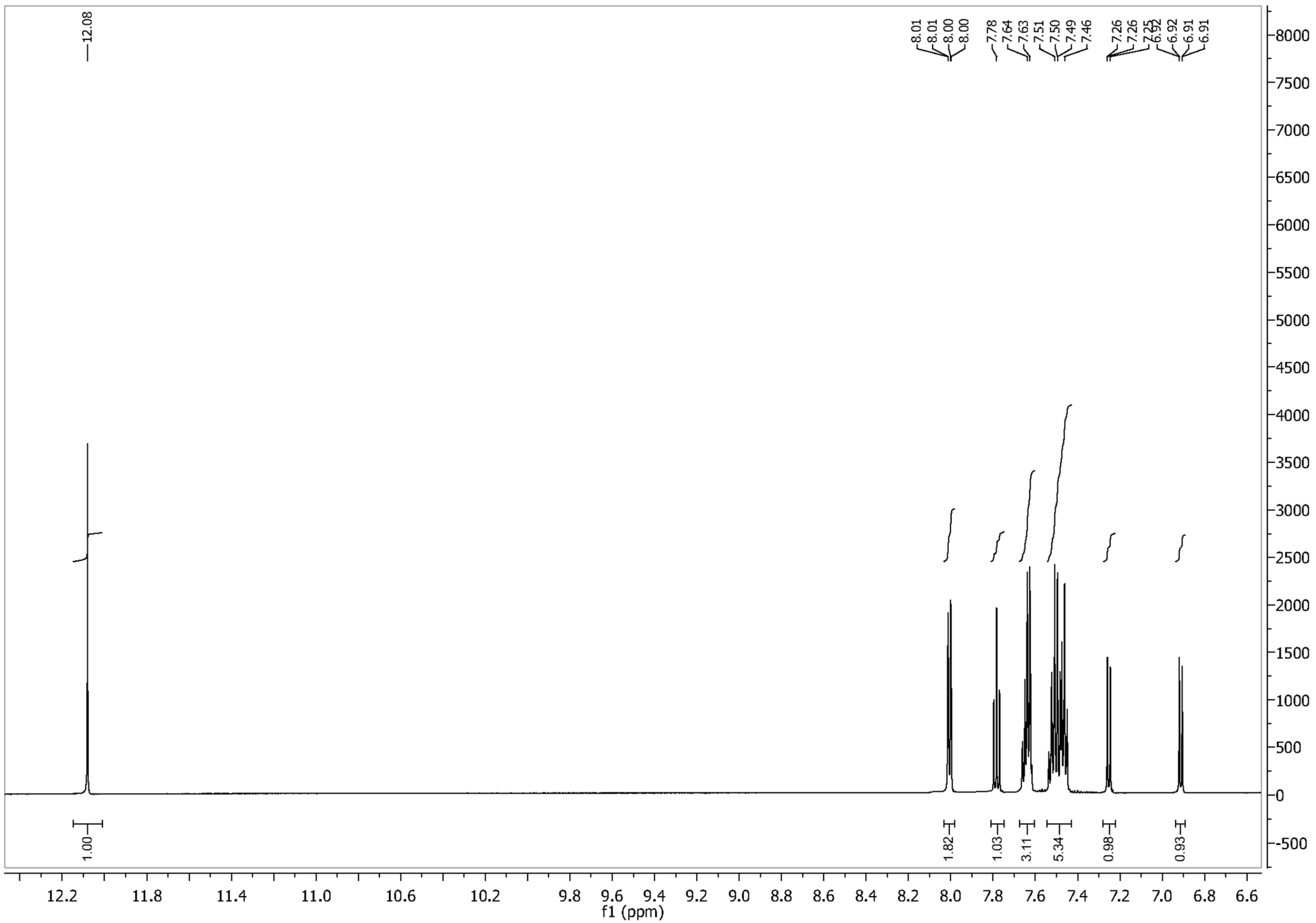

Figure S1 ^{1}H-NMR spectrum of compound **2a**

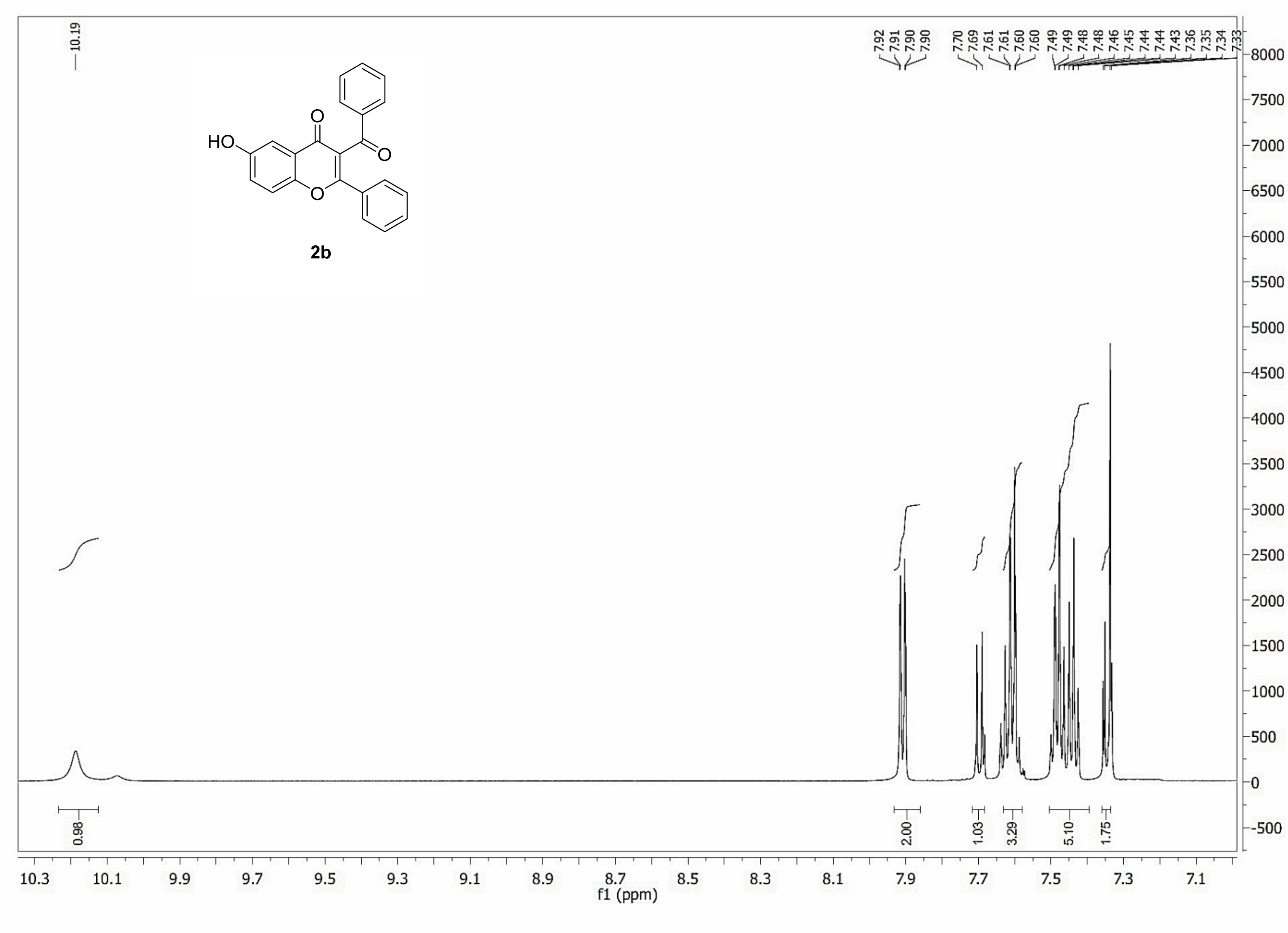

Figure S2 ^{1}H-NMR spectrum of compound **2b**

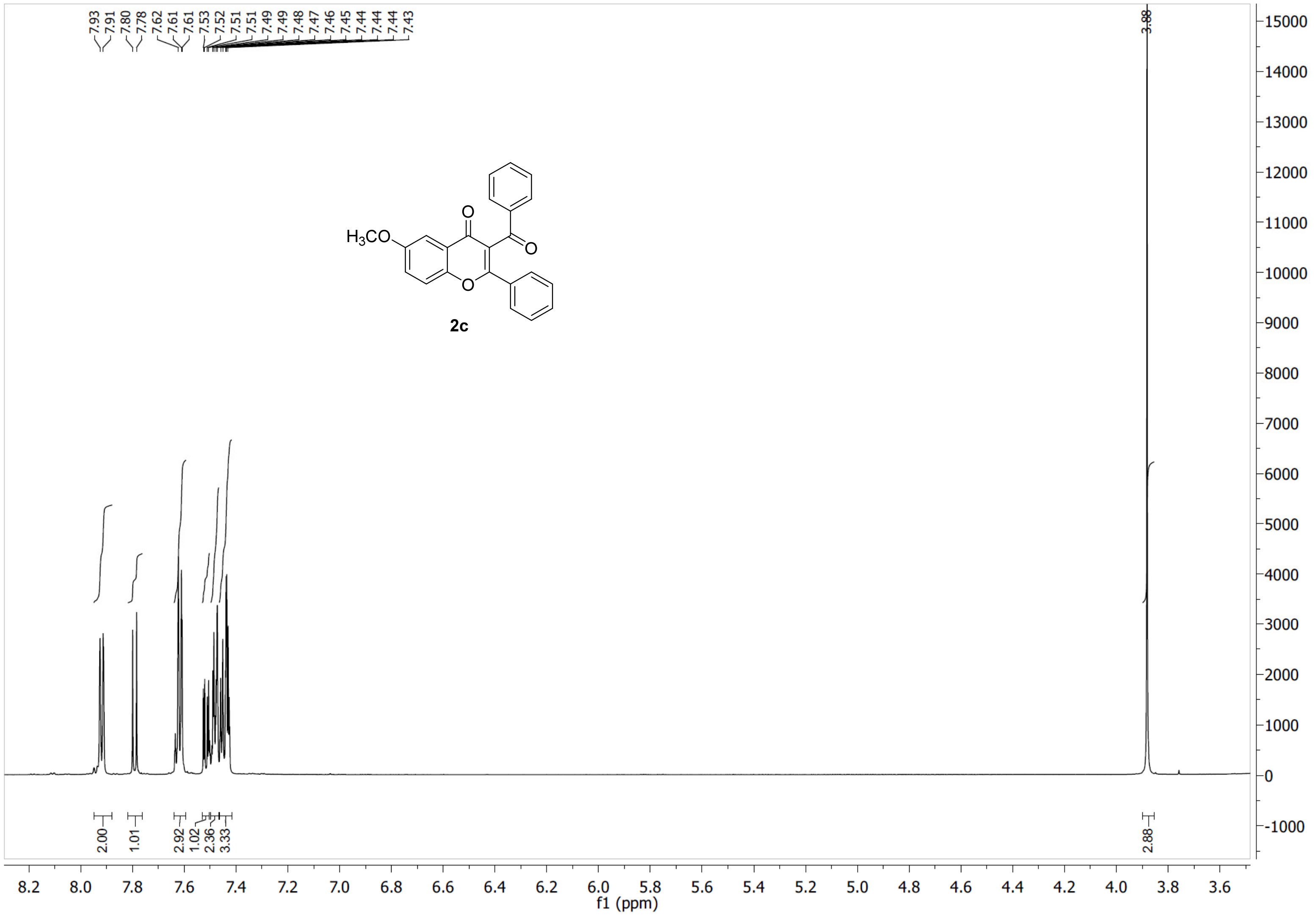

Figure S3 ^{1}H-NMR spectrum of compound **2c**

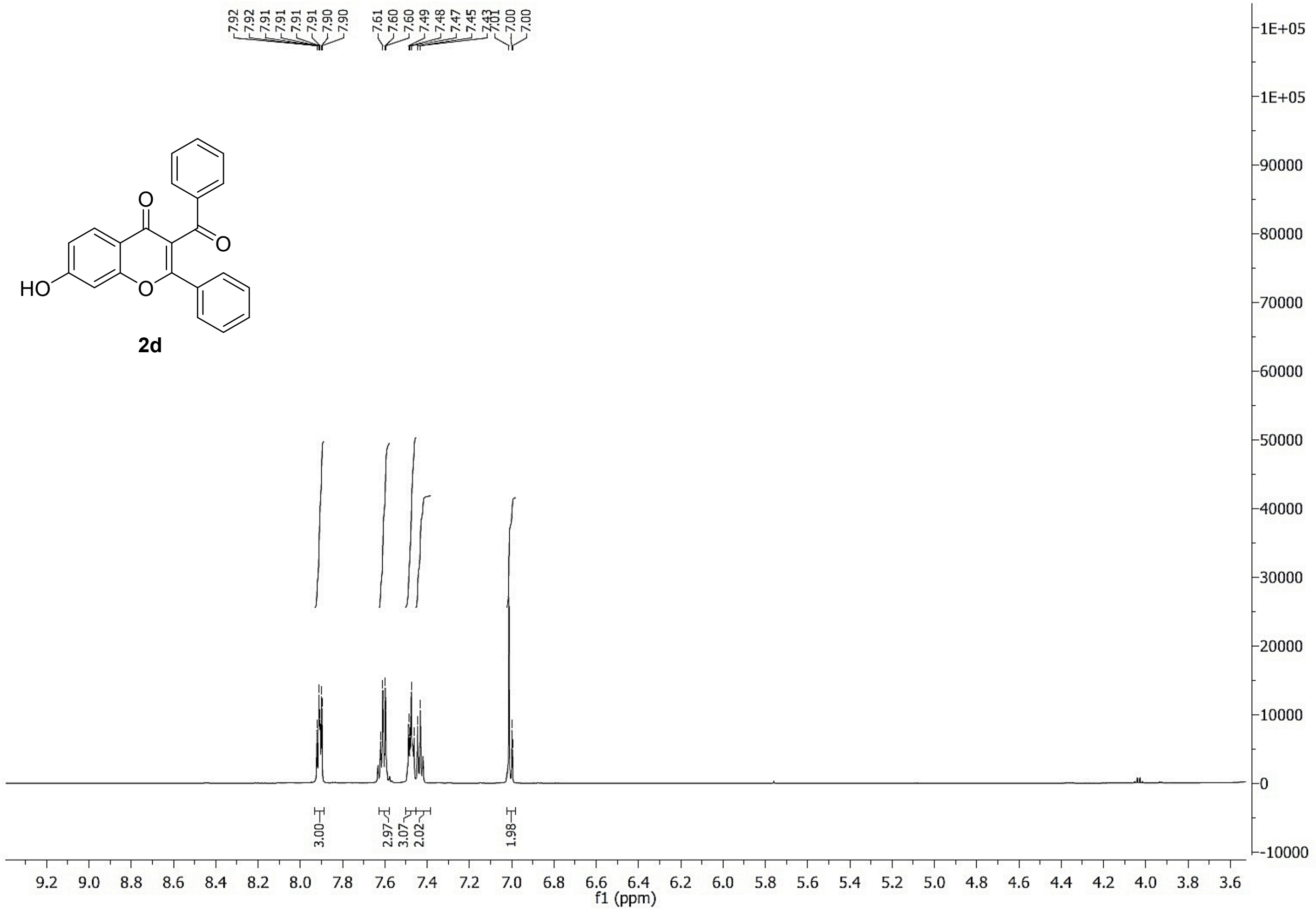

Figure S4 ^{1}H-NMR spectrum of compound **2d**

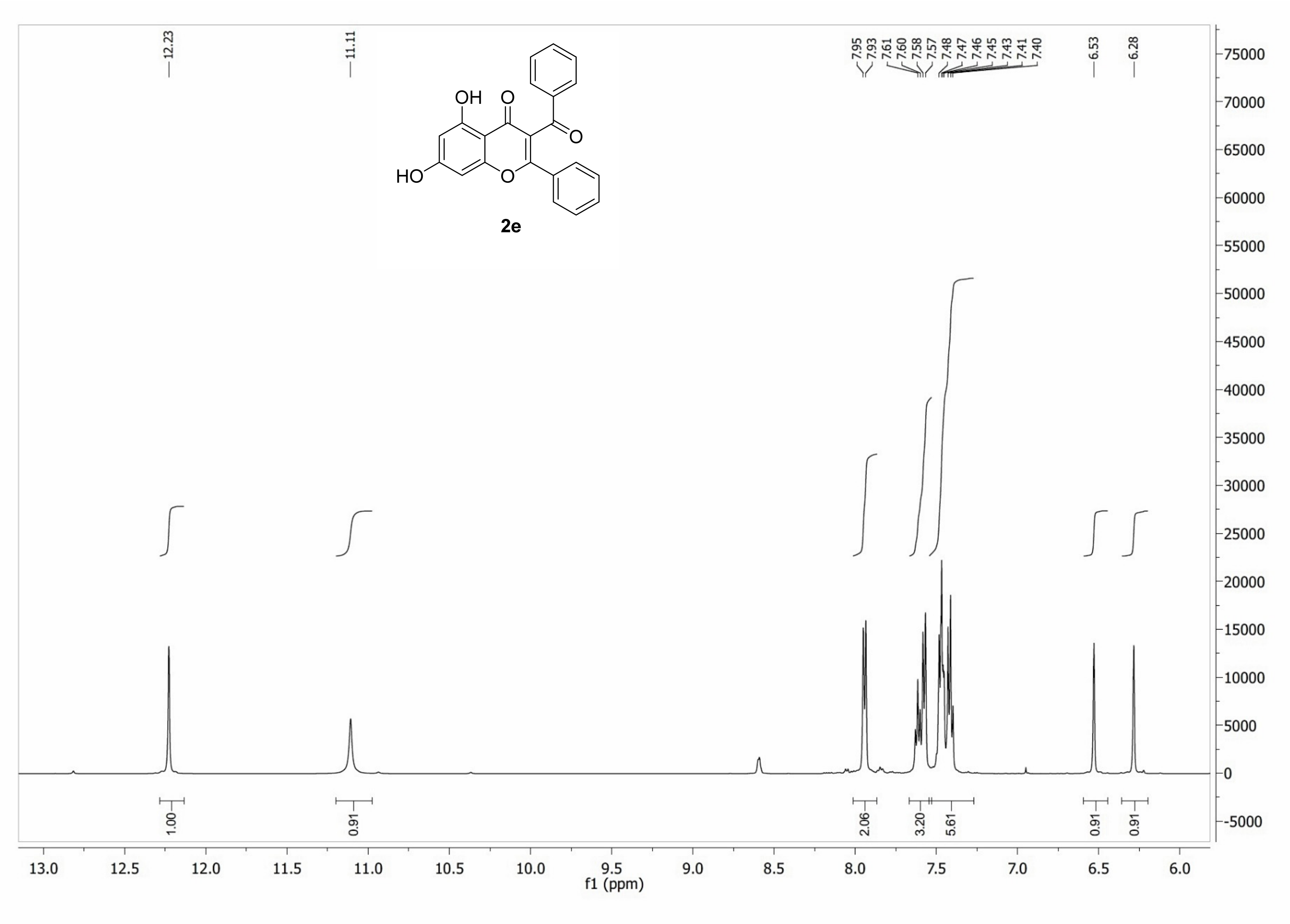

Figure S5 ^{1}H-NMR spectrum of compound **2e**

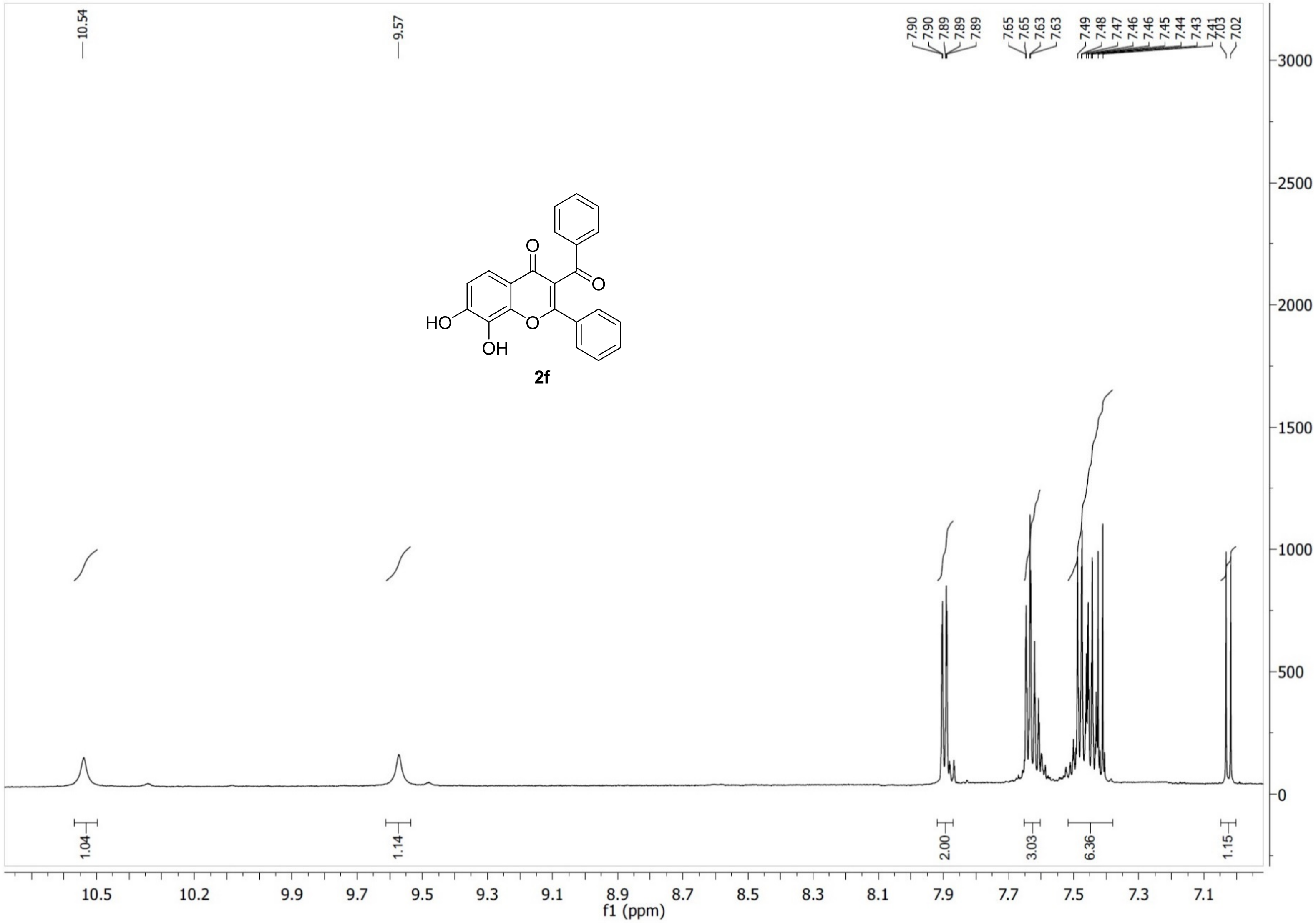

Figure S6 ^{1}H-NMR spectrum of compound **2f**

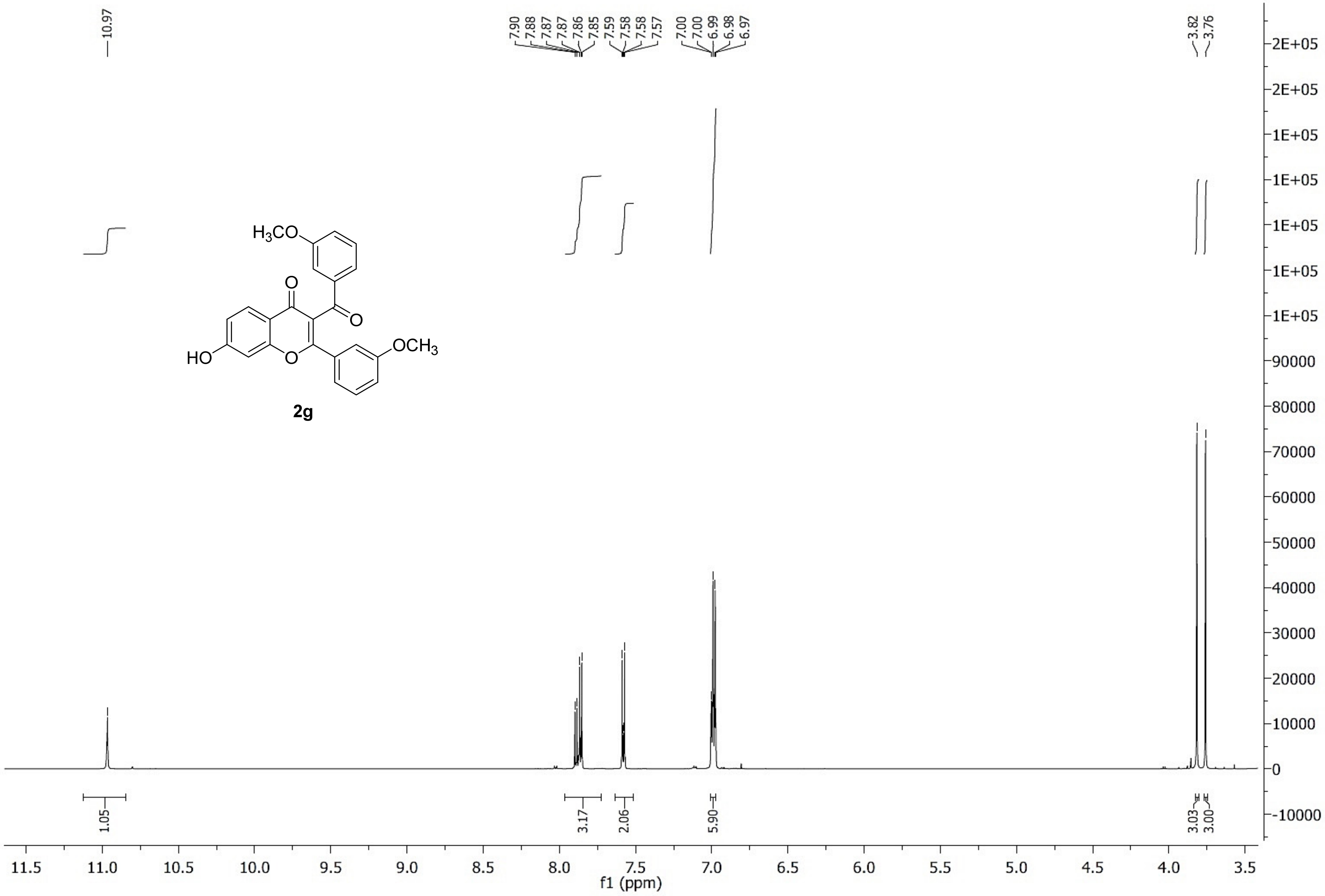

Figure S7 ^{1}H-NMR spectrum of compound **2g**

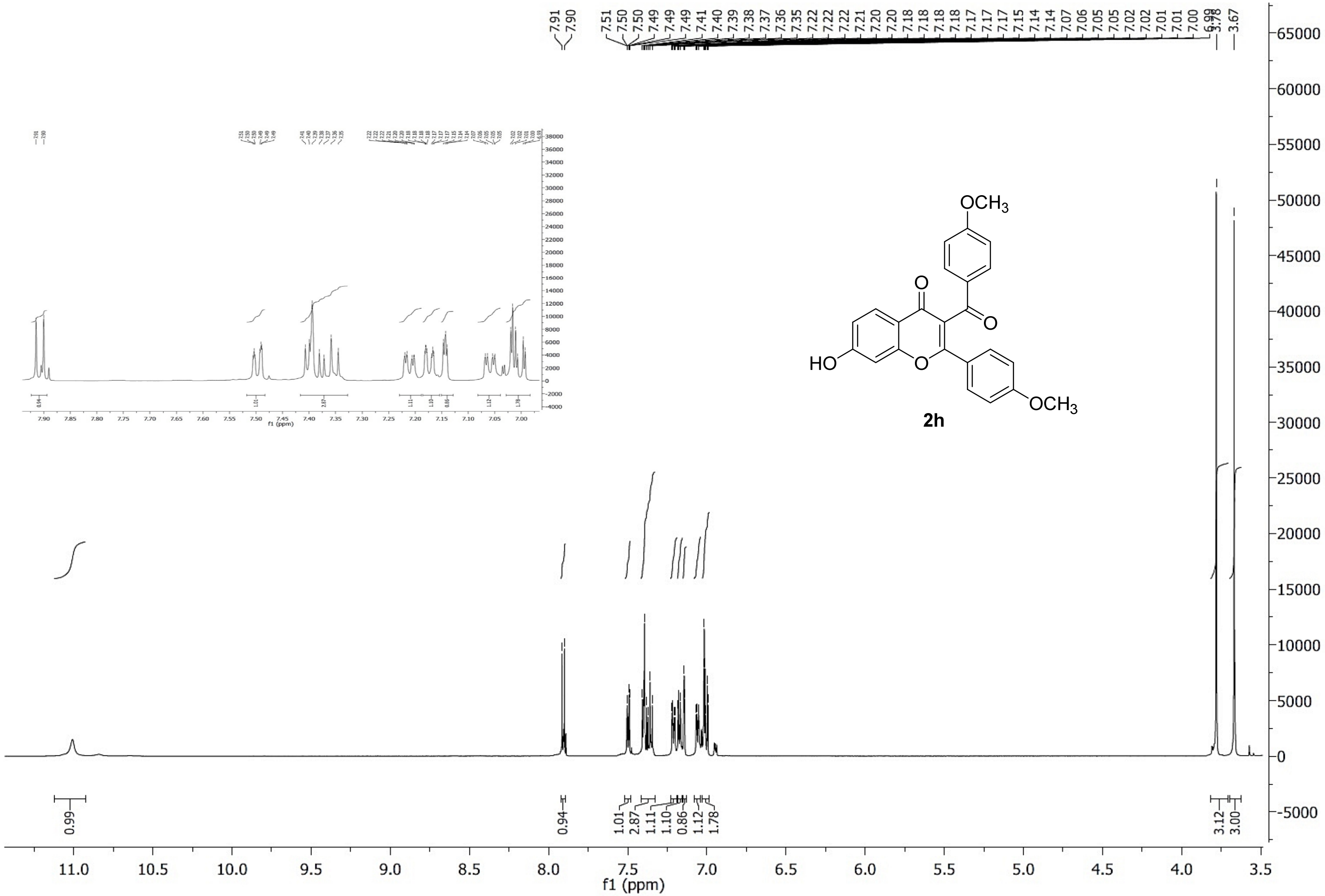

Figure S8 ^{1}H-NMR spectrum of compound **2h**

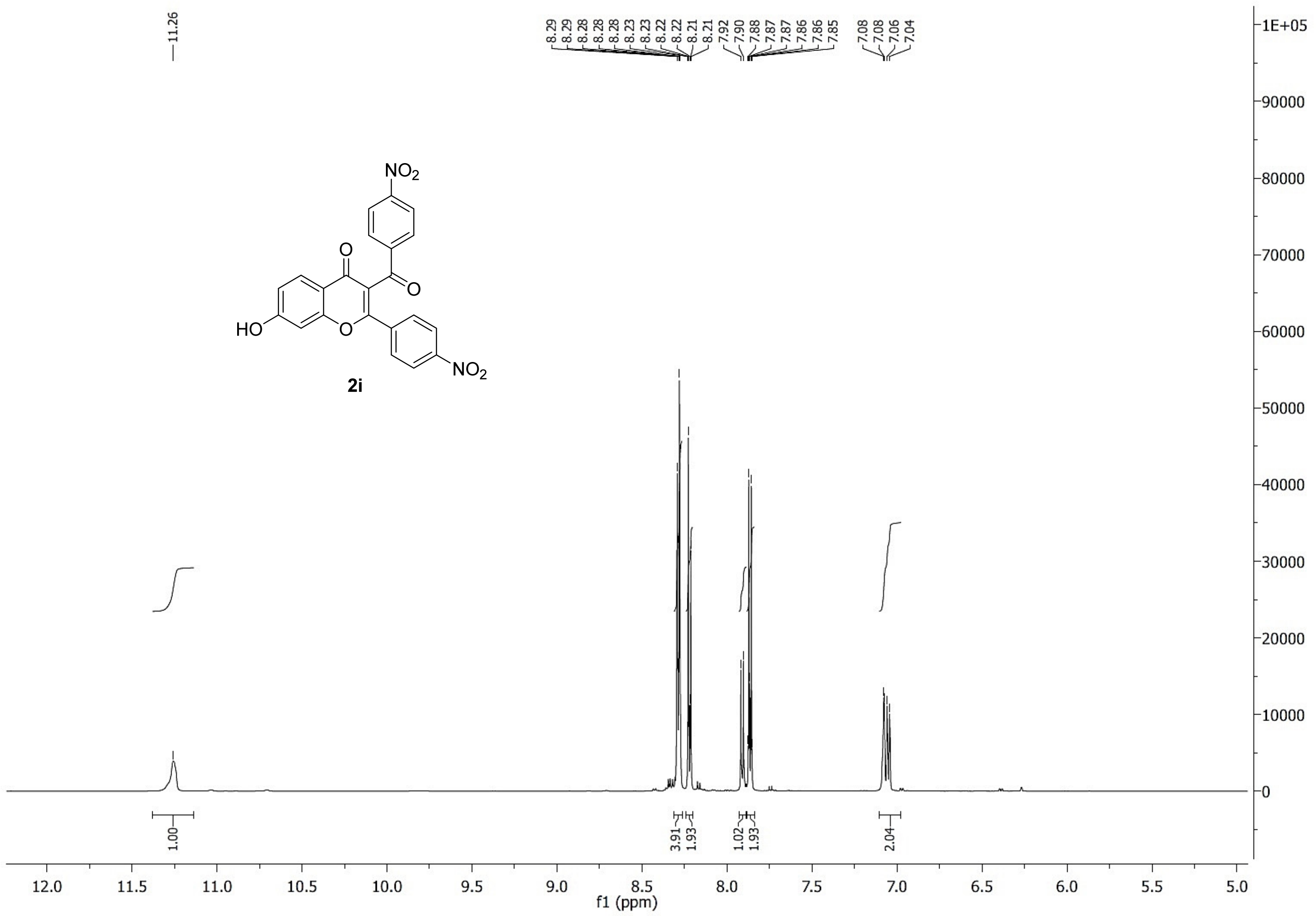

Figure S9 ^{1}H-NMR spectrum of compound **2i**

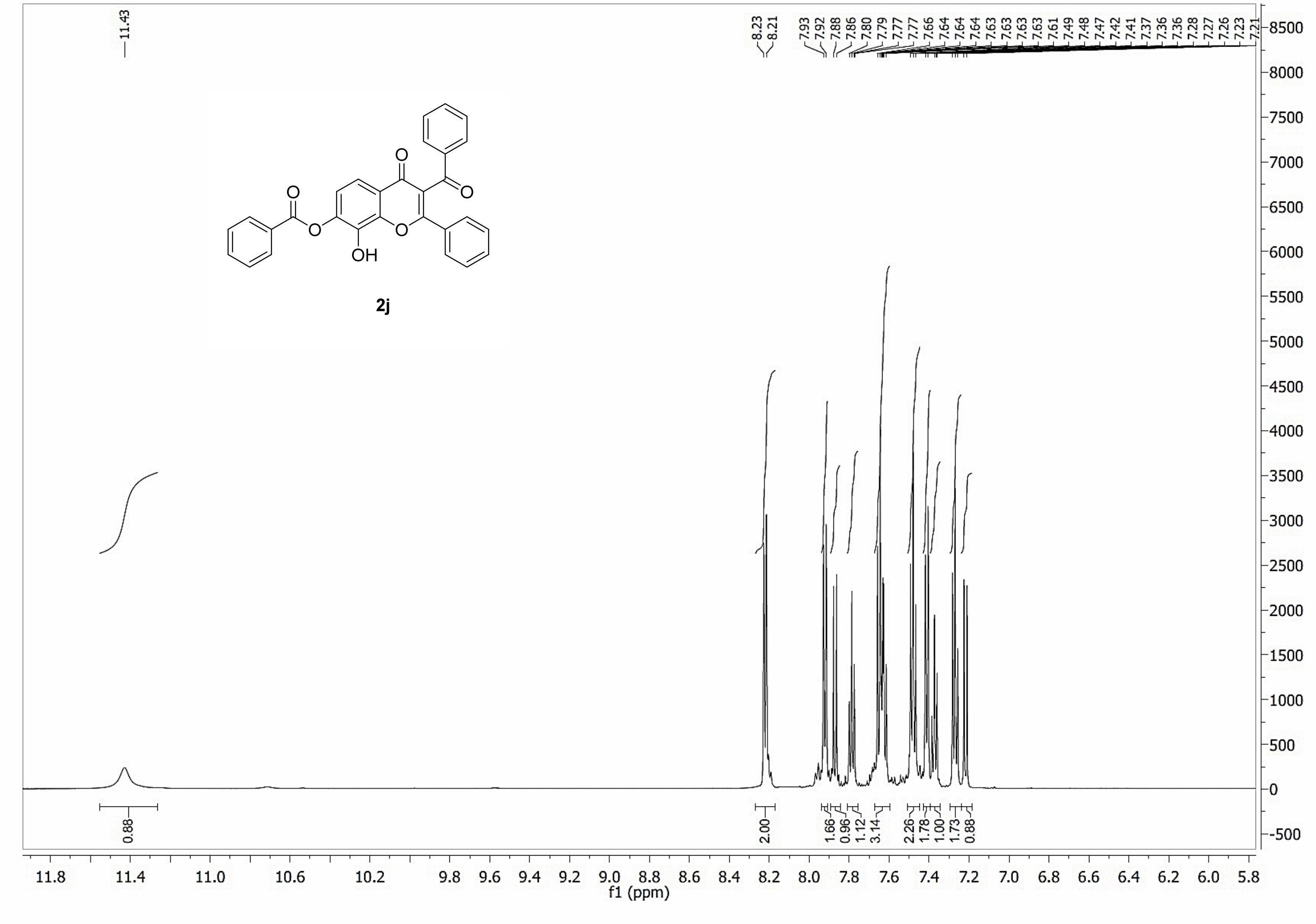

Figure S10 ^{1}H-NMR spectrum of compound **2j**

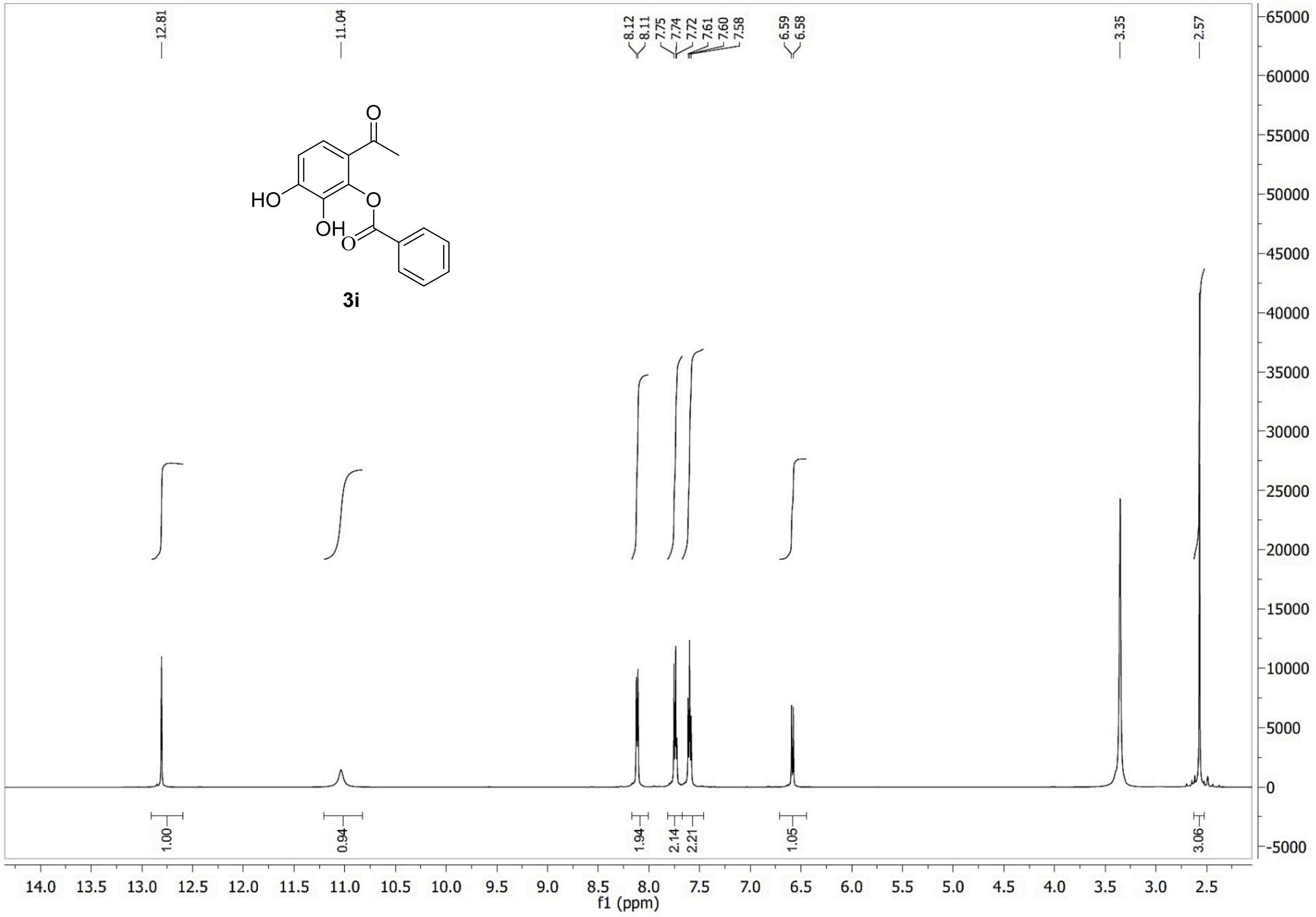

Figure S11 ^{1}H-NMR spectrum of compound **3i**

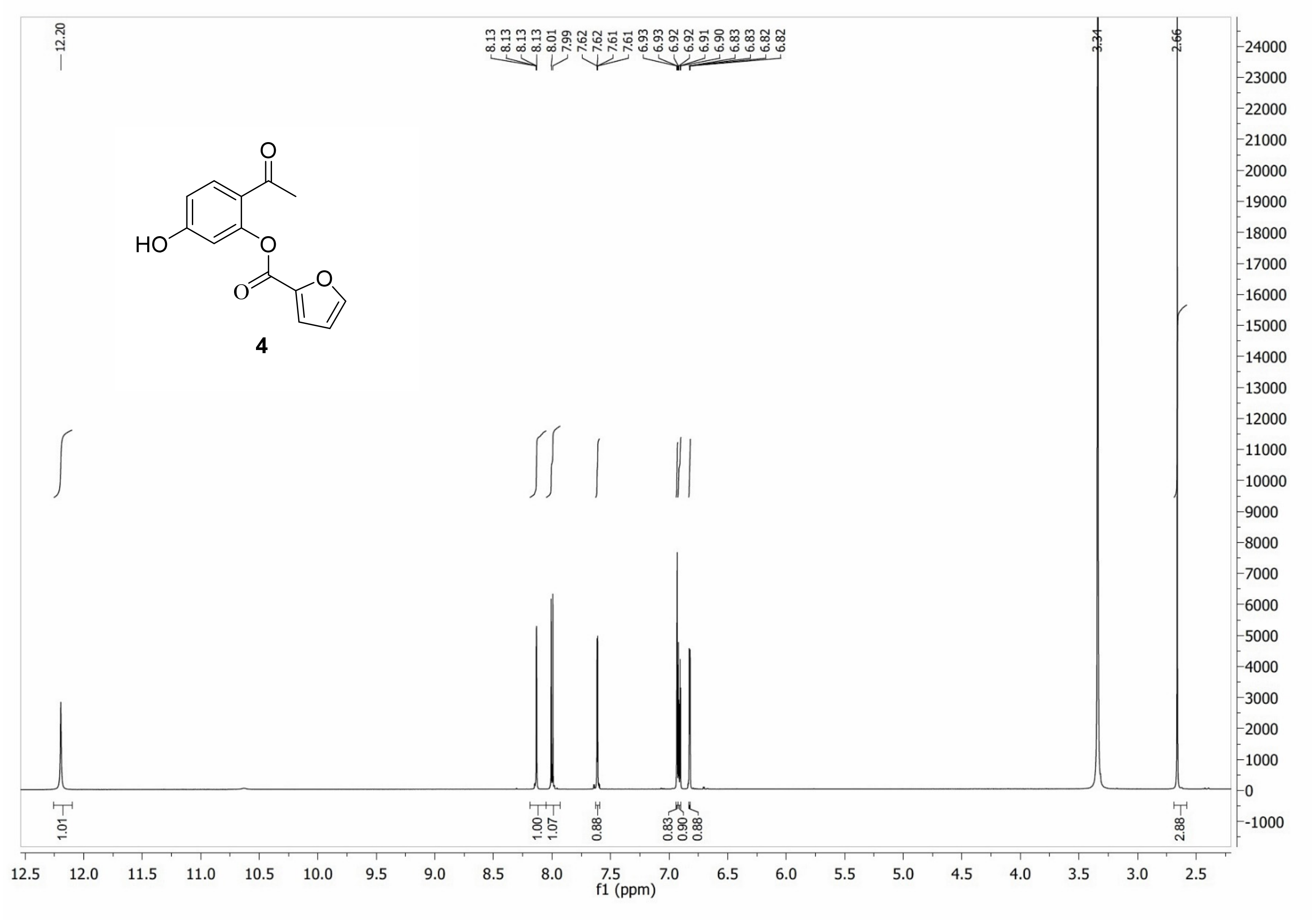

Figure S12 ^{1}H-NMR spectrum of compound **4**

3. ^{13}C-NMR spectrum of synthetic compounds

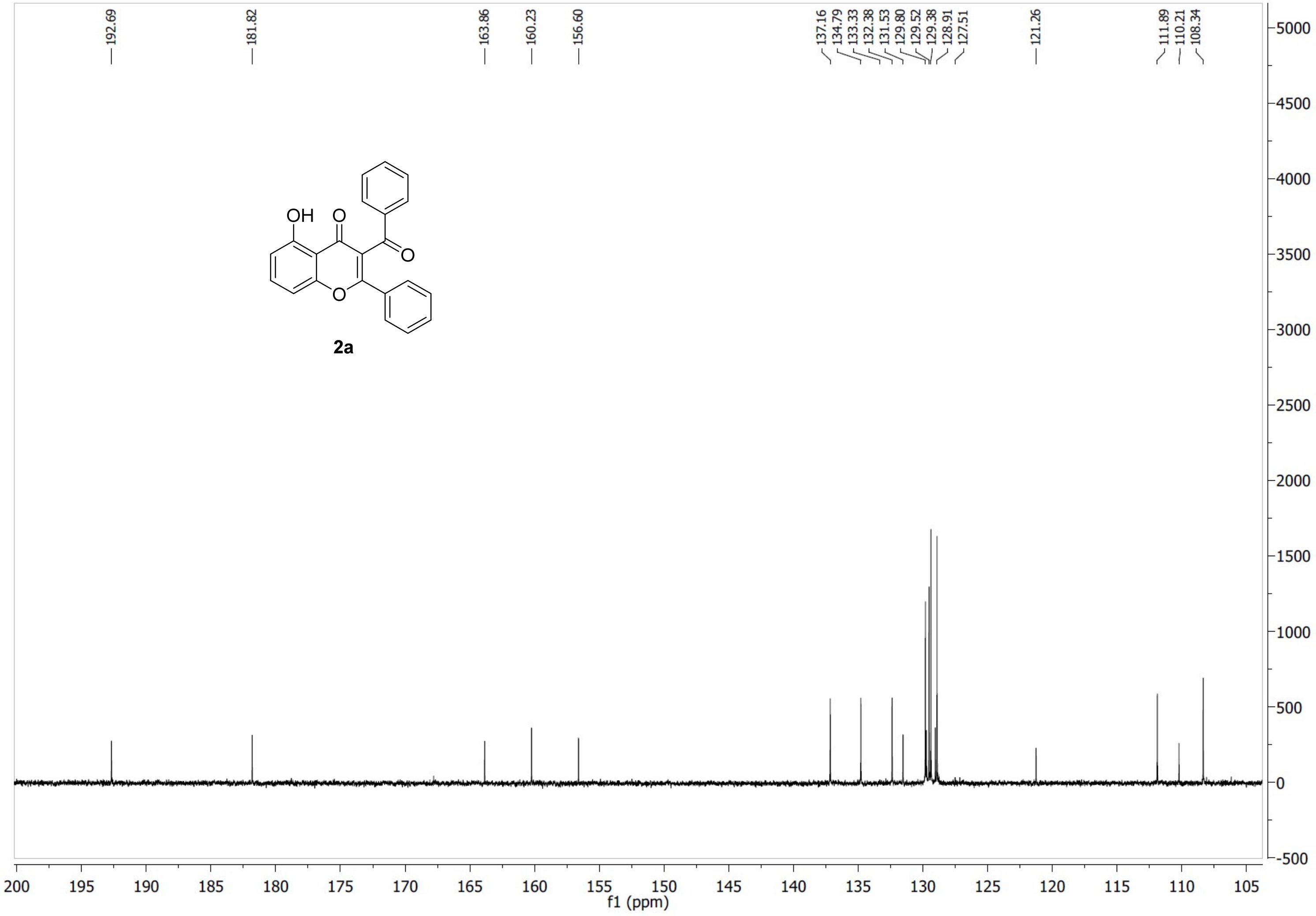

Figure S13 ^{13}C-NMR spectrum of compound **2a**

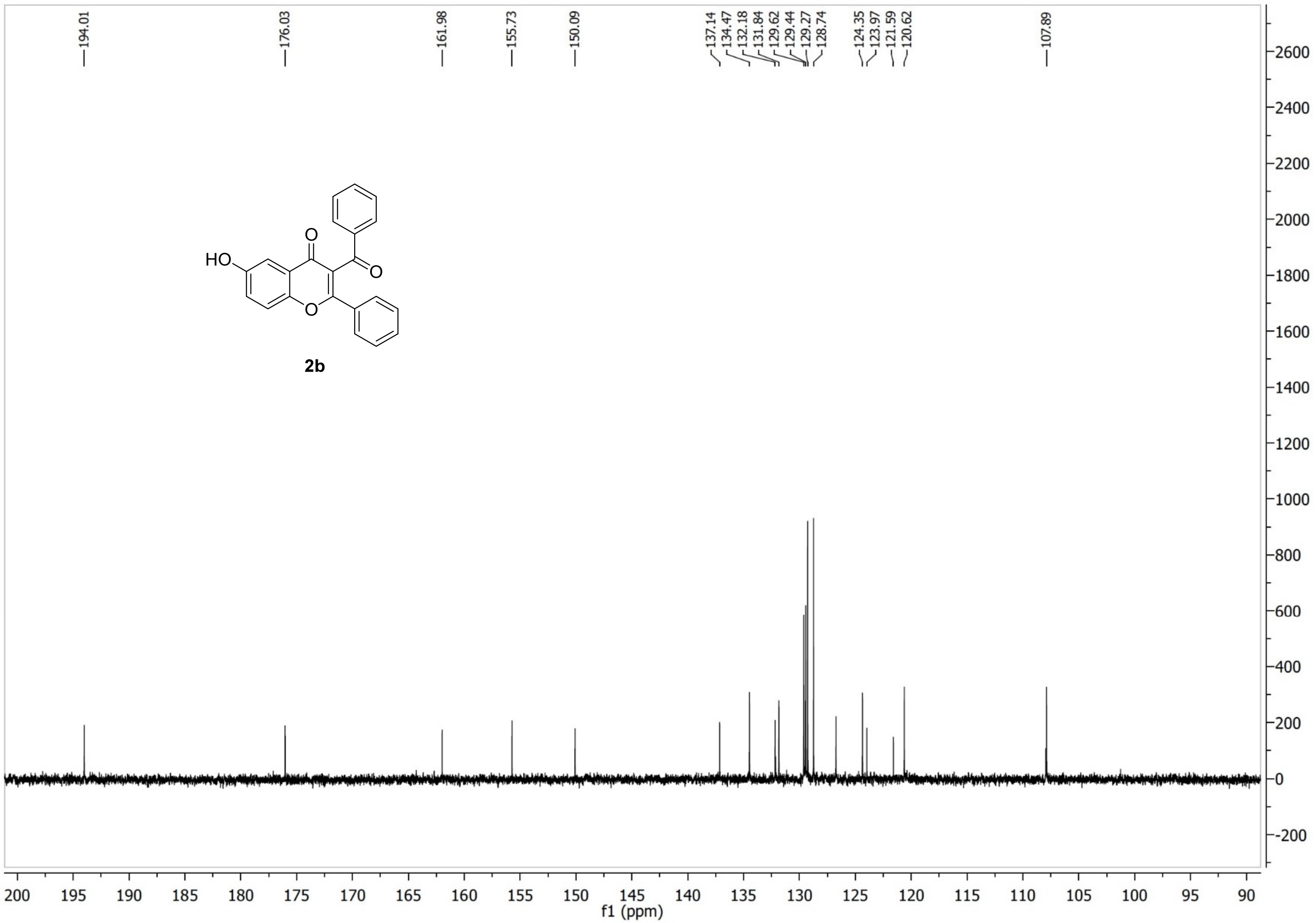

Figure S14 ^{13}C-NMR spectrum of compound **2b**

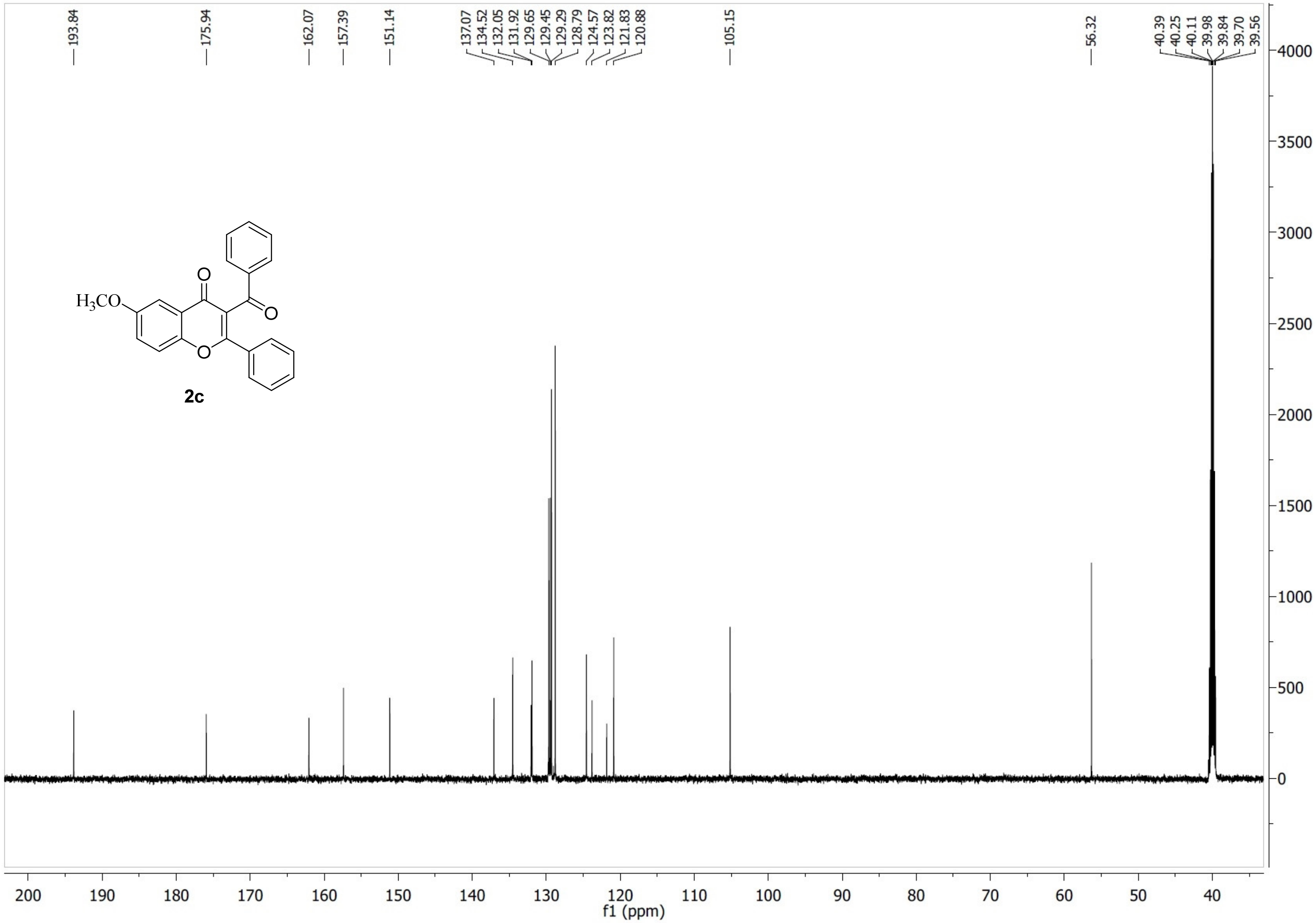

Figure S15 ^{13}C-NMR spectrum of compound **2c**

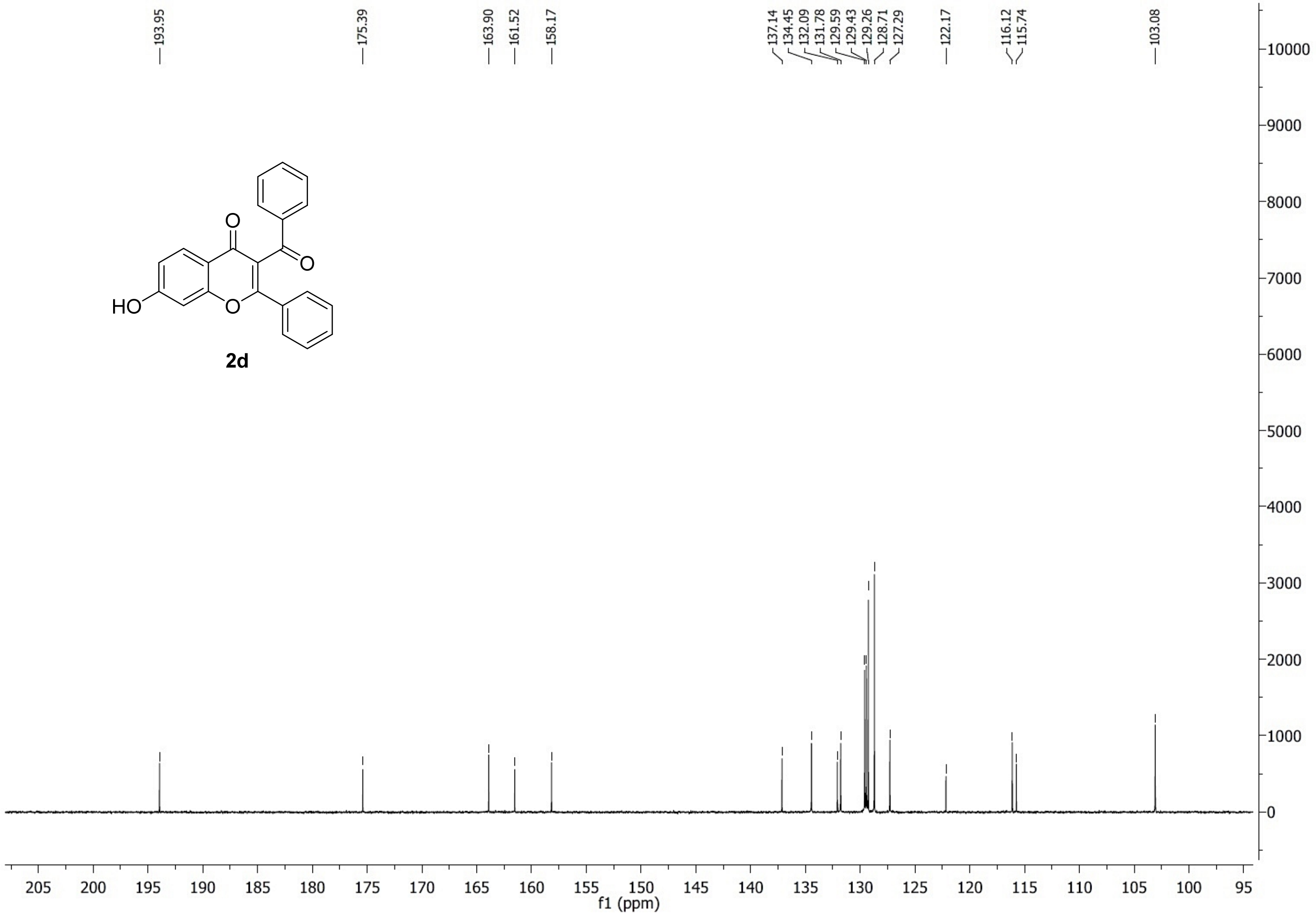

Figure S16 ^{13}C-NMR spectrum of compound **2d**

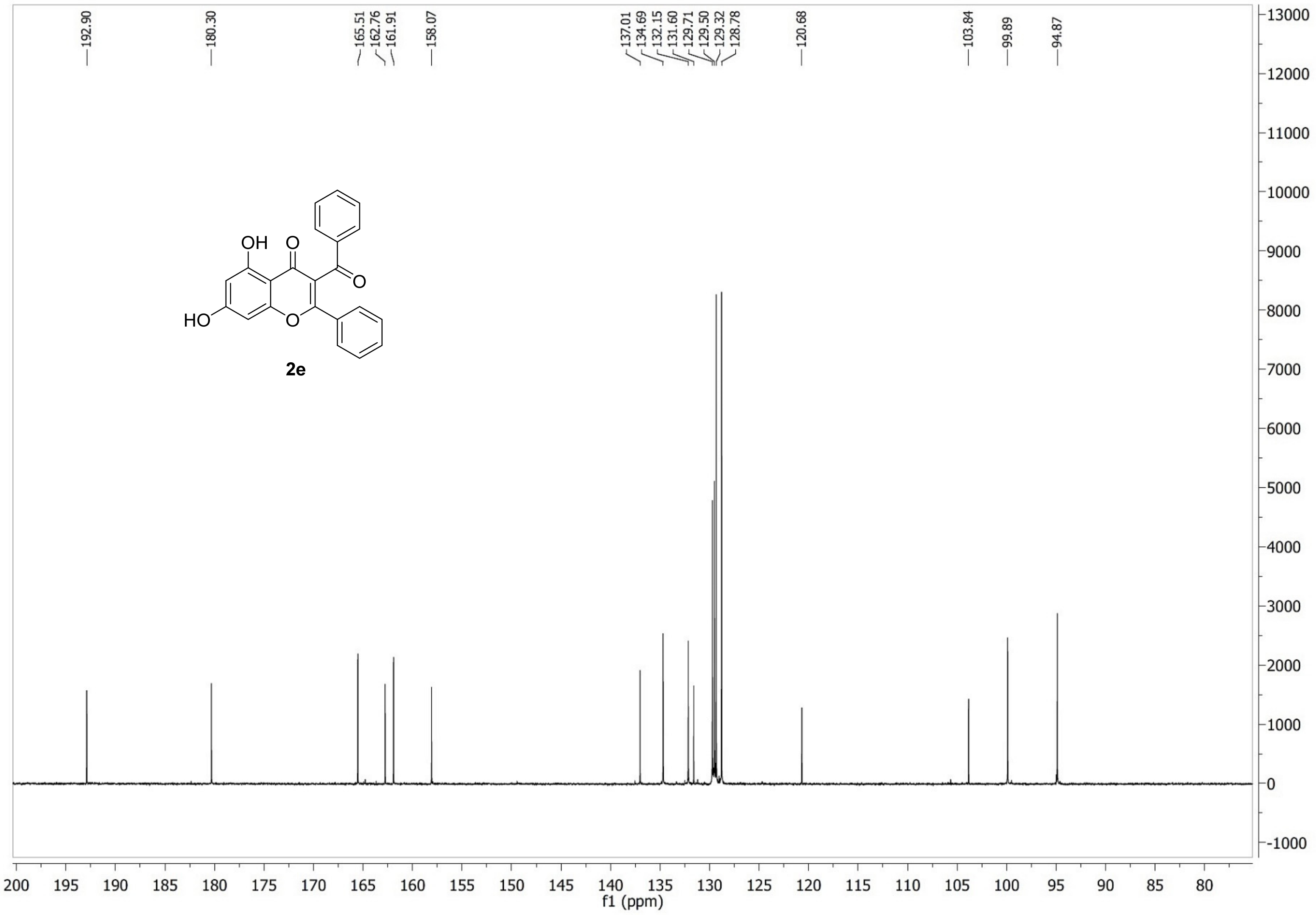

Figure S17 ^{13}C-NMR spectrum of compound **2e**

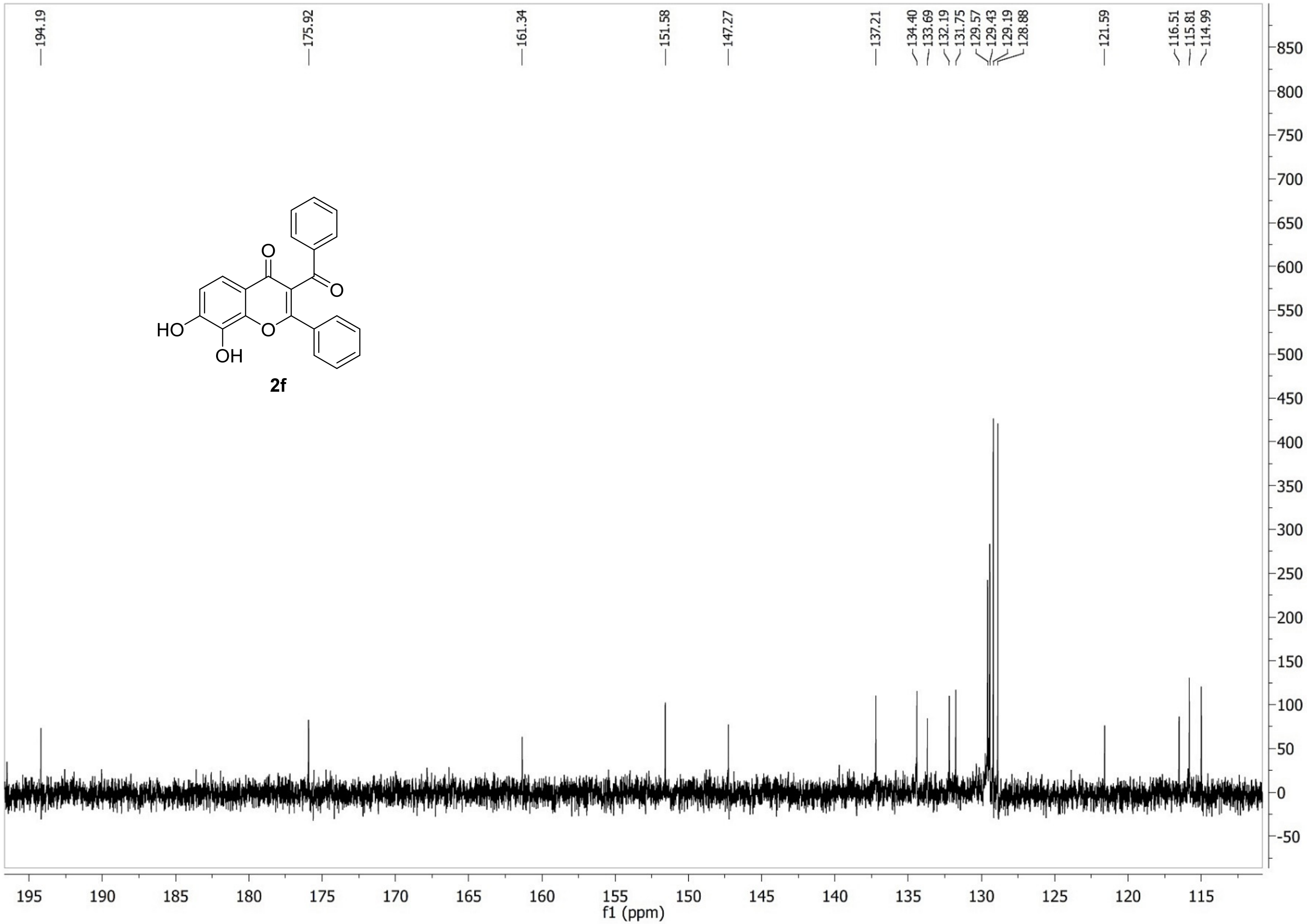

Figure S18 ^{13}C-NMR spectrum of compound **2f**

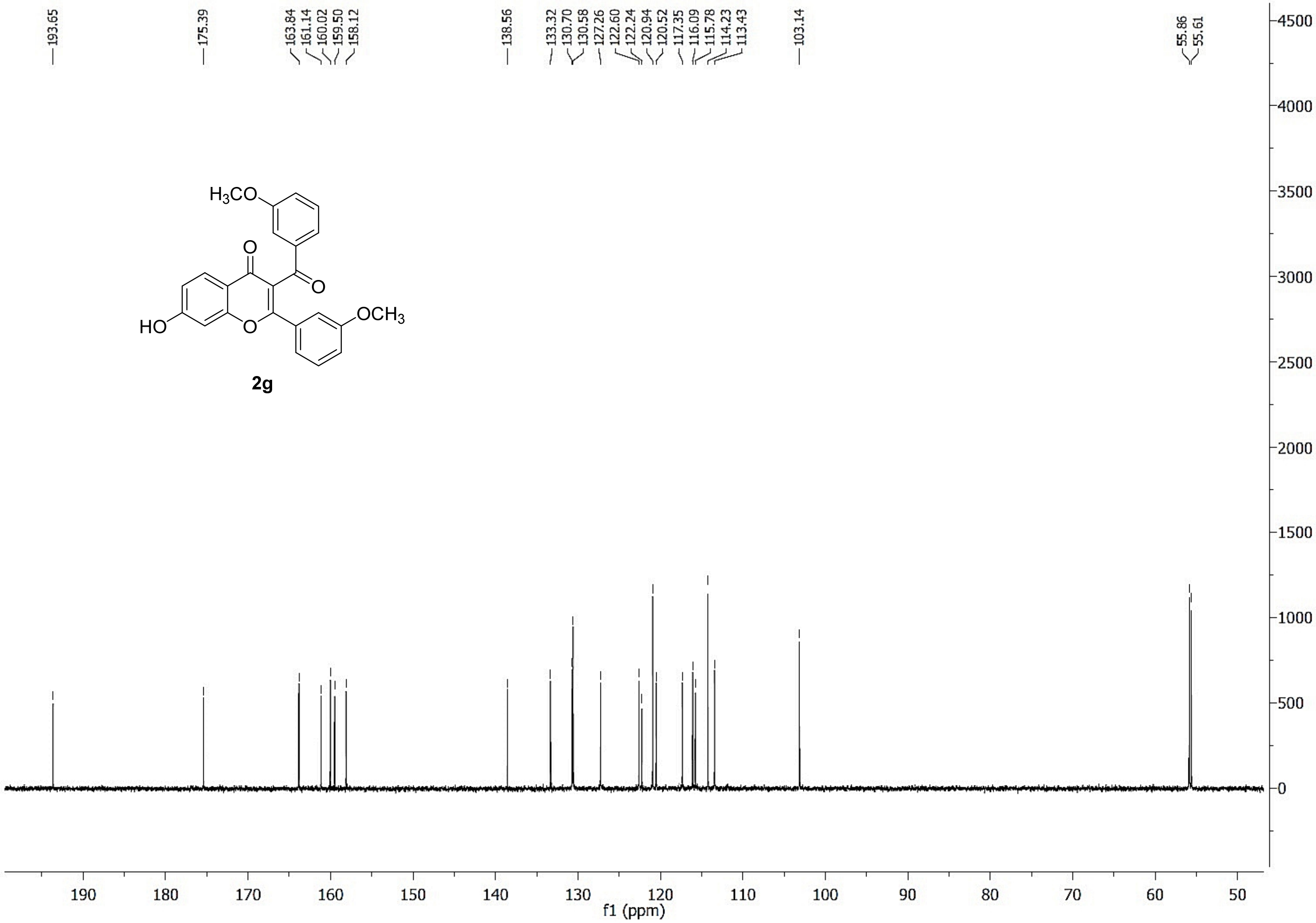

2g

Figure S19 ^{13}C-NMR spectrum of compound **2g**

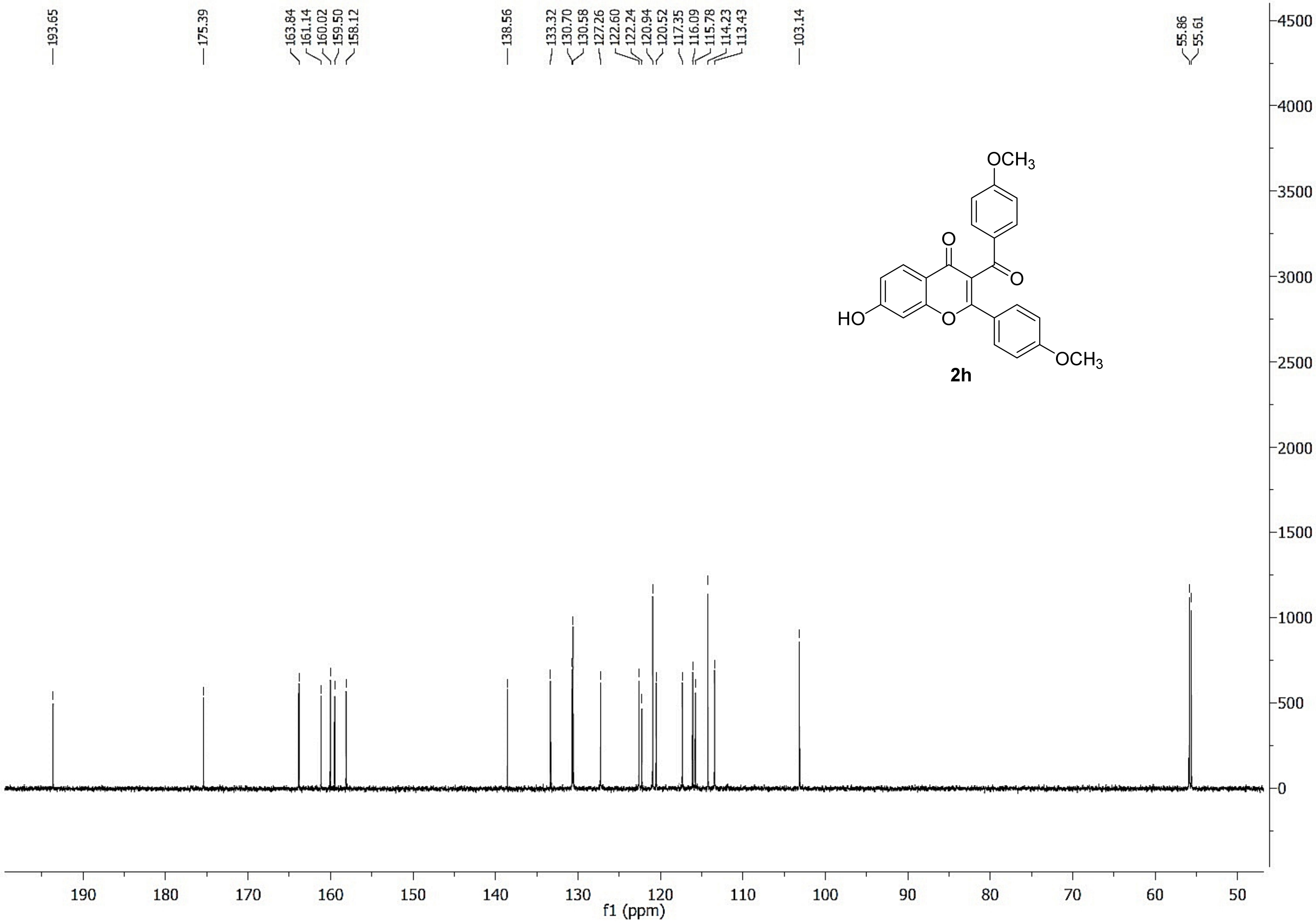

Figure S20 ^{13}C-NMR spectrum of compound **2h**

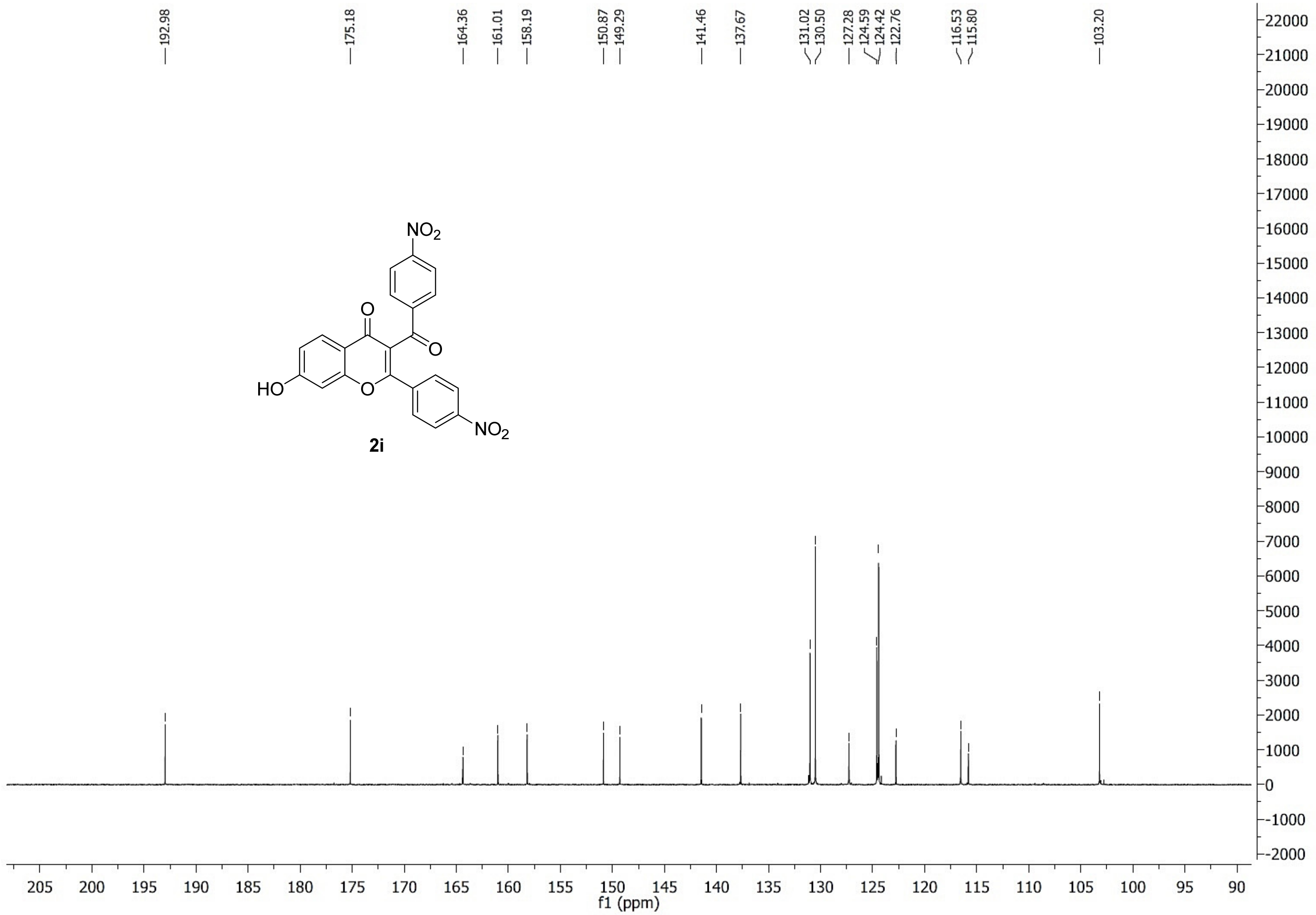

Figure S21 ^{13}C-NMR spectrum of compound **2i**

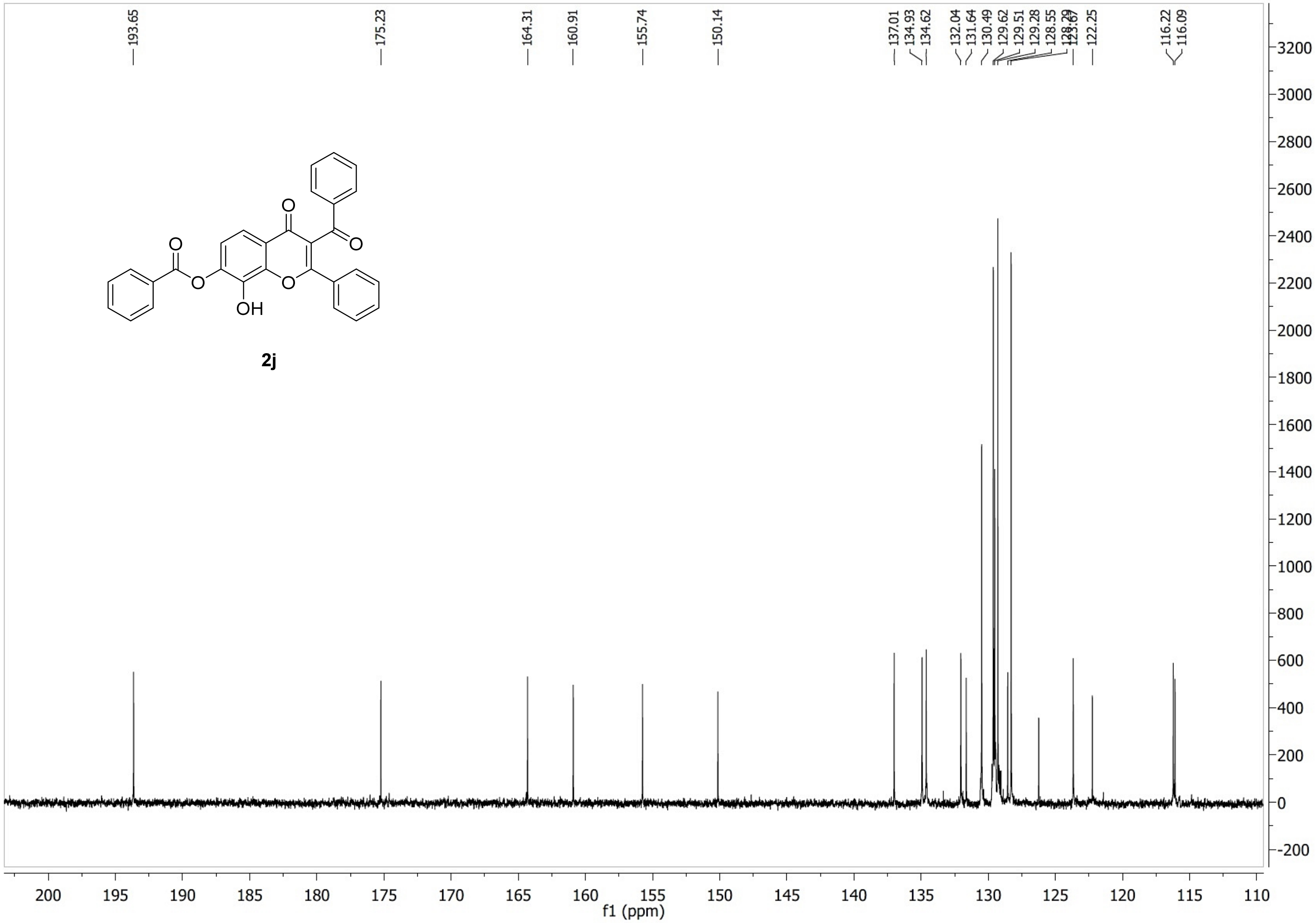

Figure S22 ^{13}C-NMR spectrum of compound **2j**

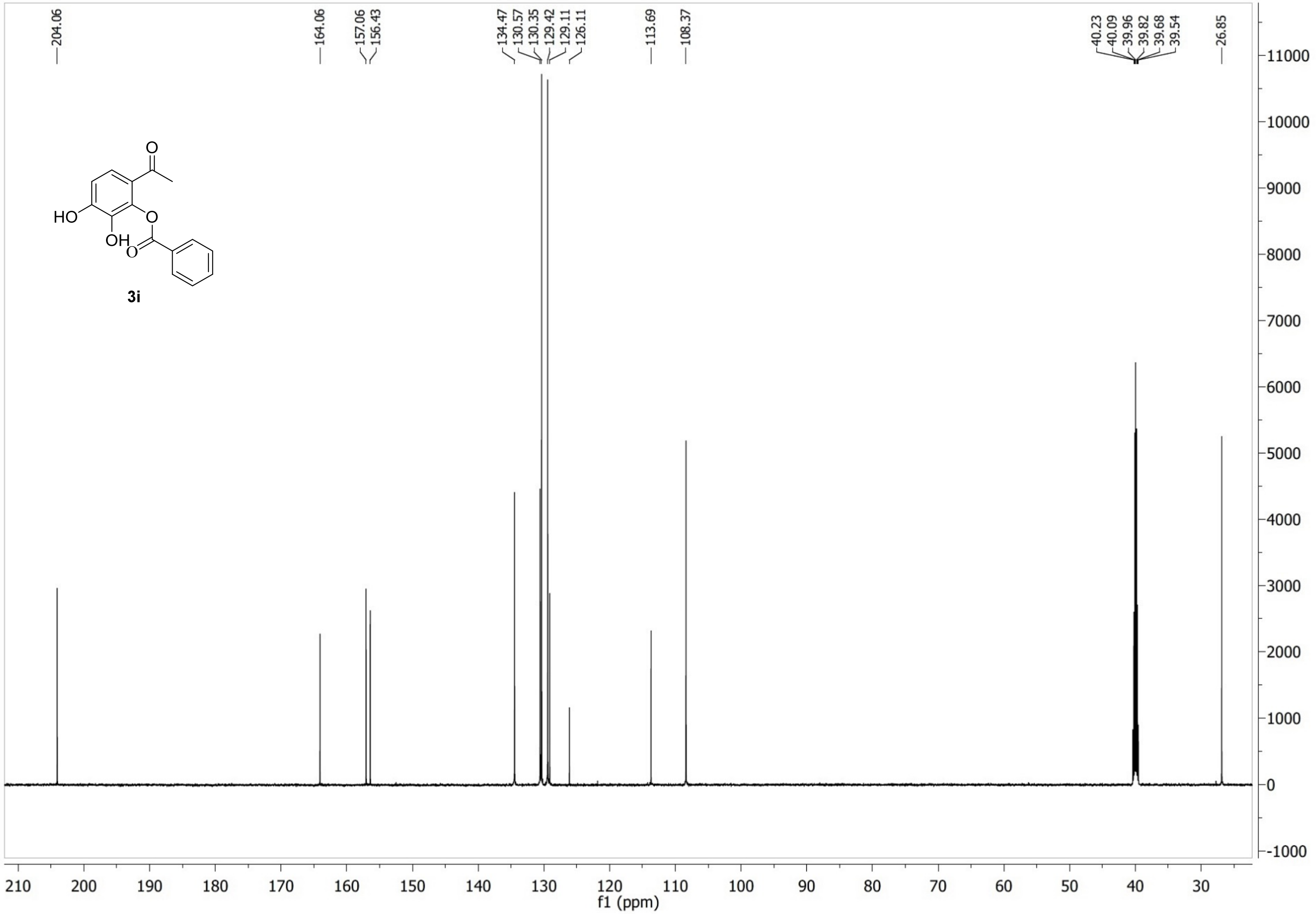

Figure S23 ^{13}C-NMR spectrum of compound **3i**

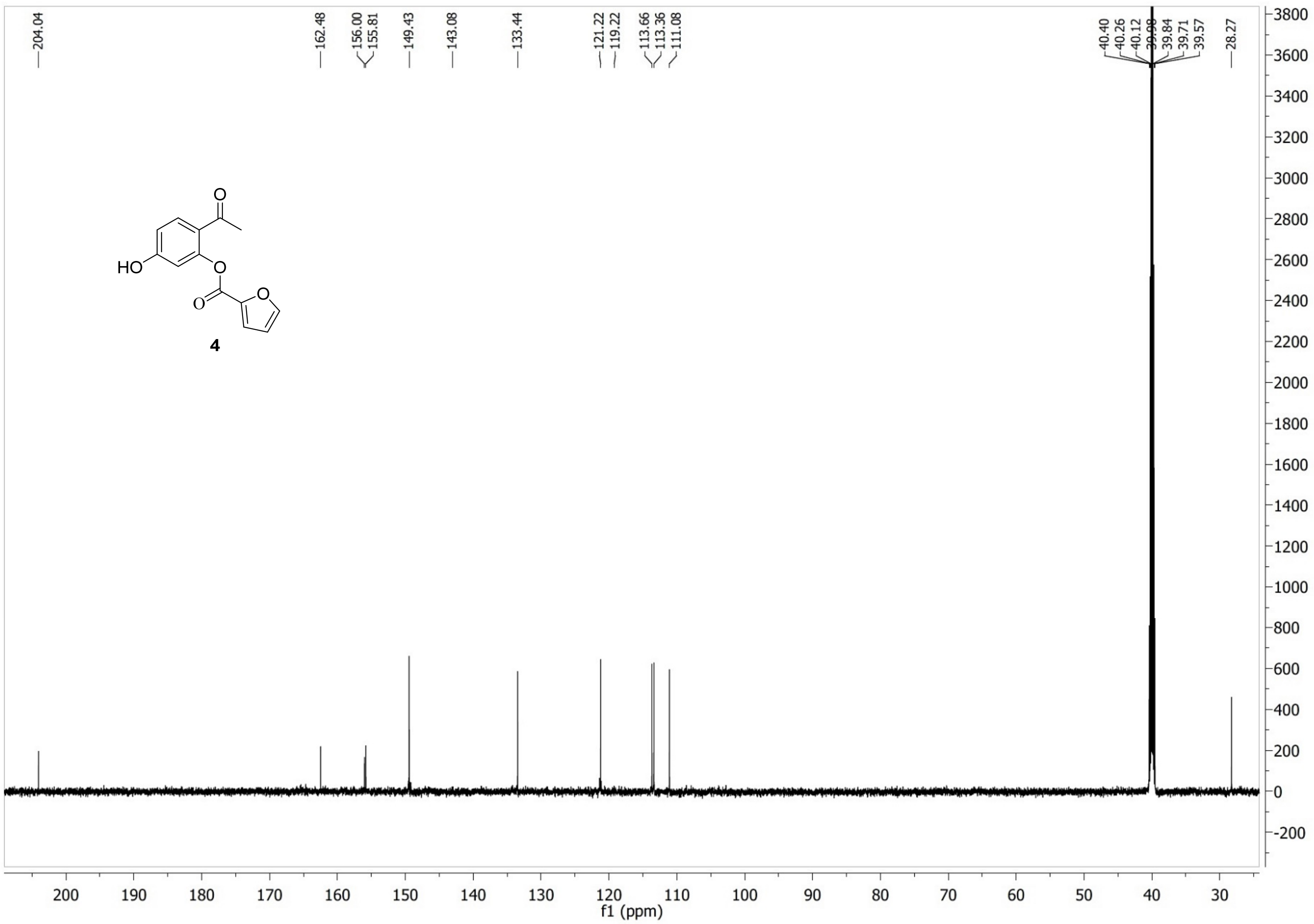

Figure S24 ^{13}C-NMR spectrum of compound **4**

4. LCMS spectrum of synthetic compounds

Mass spectroscopy condition

The LC–ESI–MS/QTOF-MS system consists of HPLC unit 1260 infinity series (Agilent Technologies, Waldbronn, Germany) coupled with an ultra-high-definition accurate mass spectrometer (Agilent Technologies, Singapore). One microliter was injected to the system at a flow rate of 0.5 mL/min. The column temperature was maintained at 35 °C. The mobile phase consisted of A (0.1% formic acid in type I water, v/v) and B (0.1% formic acid in acetonitrile, v/v). The isocratic elution was 50% of mobile phase A. The dual electrospray ionization (ESI) source was operated in positive modes. The ESI condition was as follows: drying gas (gas N_2) temperature, 350 °C; gas flow rate, 10 L/min; nebulizer pressure, 30 psig; mass range, 200–800m/z; scan rate, 4 spectra/s; capillary voltage, 3500 V; skimmer voltage, 65 V; octapole RFV, 750 V; and fragment voltage, 50 V.

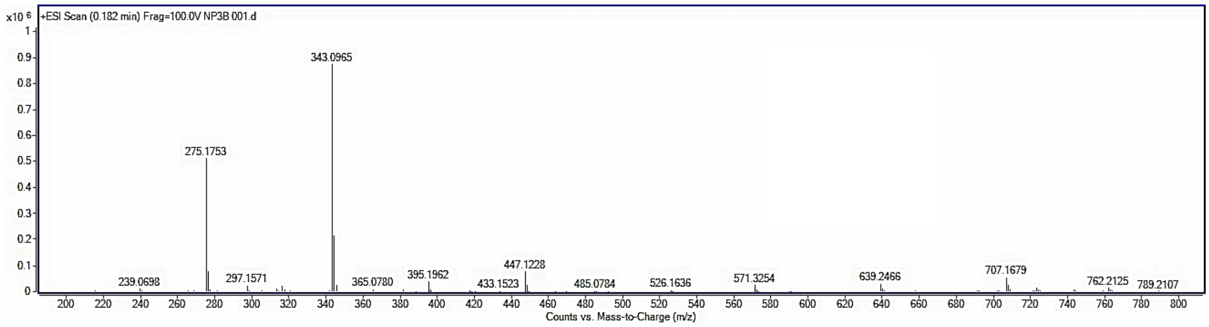

Figure S25 Mass spectrum (MS-ESI) of compound **2a**

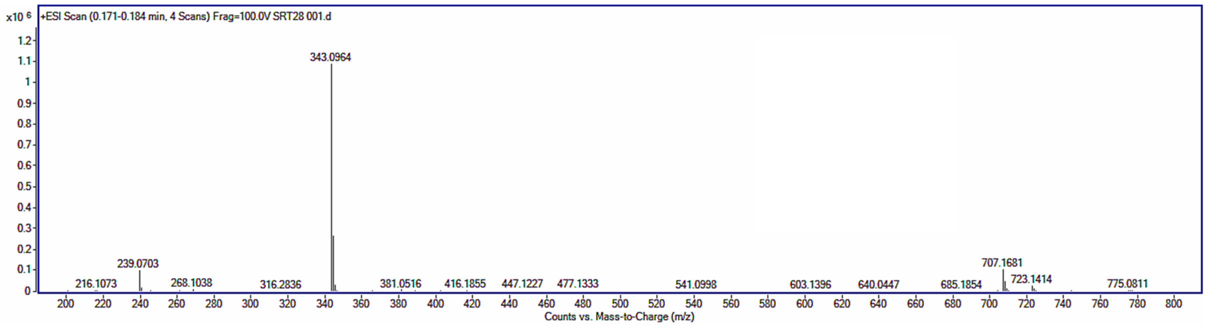

Figure S26 Mass spectrum (MS-ESI) of compound **2b**

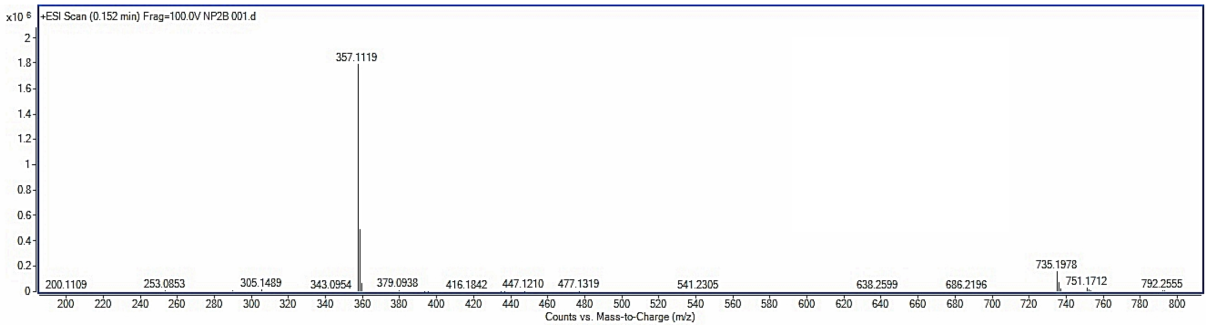

Figure S27 Mass spectrum (MS-ESI) of compound **2c**

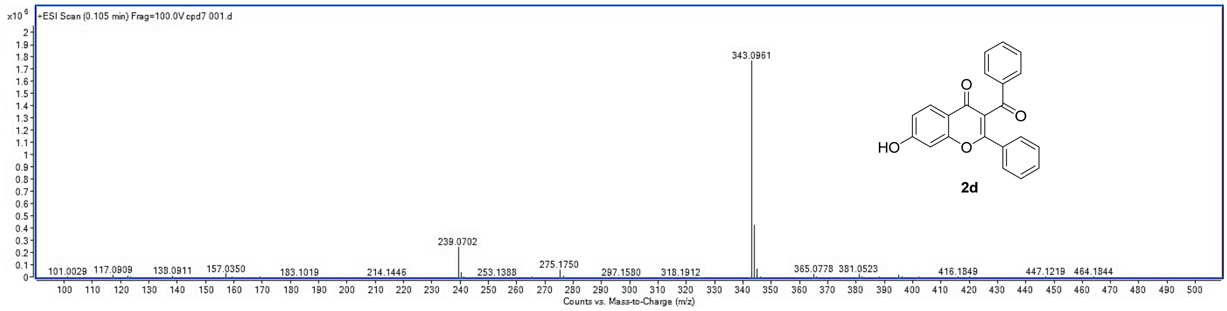

Figure S28 Mass spectrum (MS-ESI) of compound **2d**

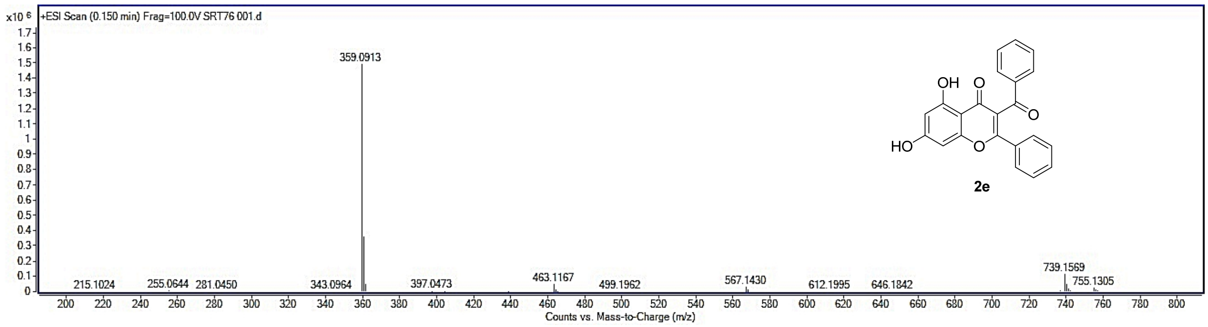

Figure S29 Mass spectrum (MS-ESI) of compound **2e**

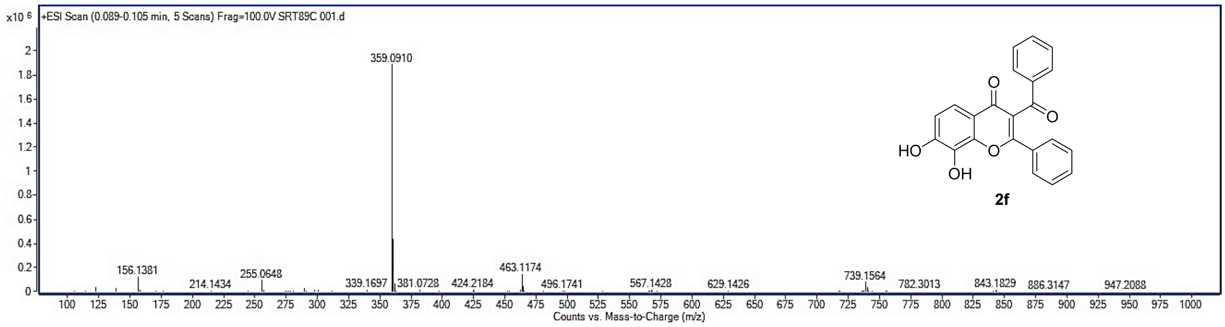

Figure S30 Mass spectrum (MS-ESI) of compound **2f**

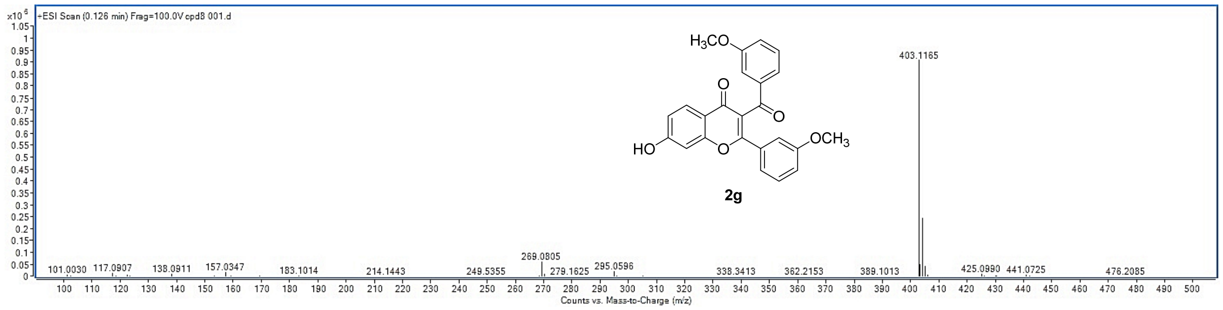

Figure S31 Mass spectrum (MS-ESI) of compound **2g**

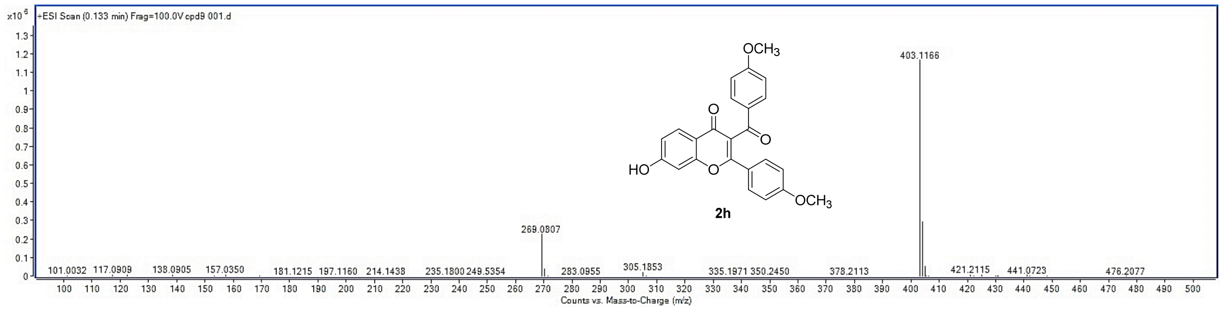

Figure S32 Mass spectrum (MS-ESI) of compound **2h**

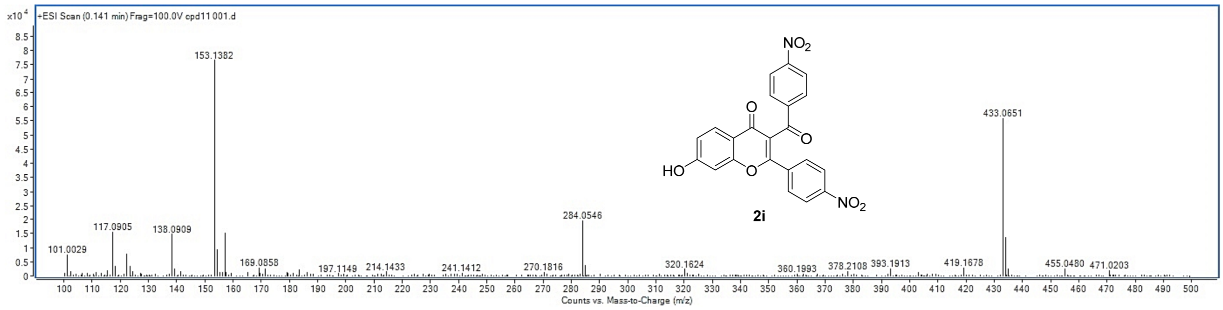

Figure S33 Mass spectrum (MS-ESI) of compound **2i**

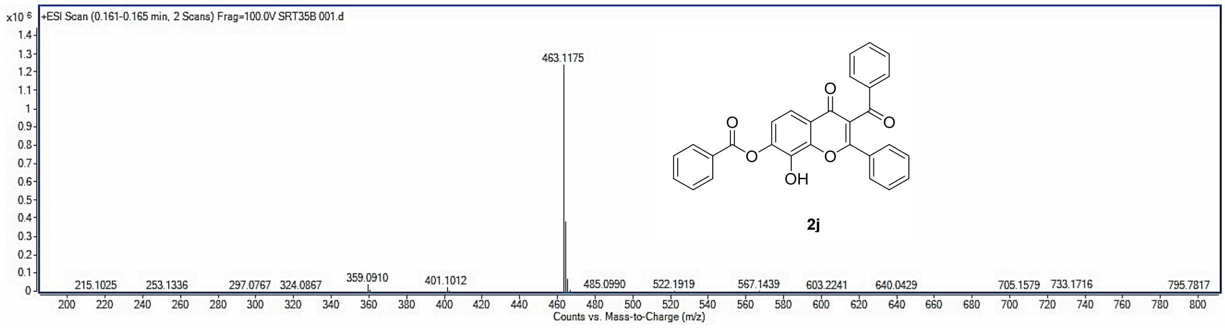

Figure S34 Mass spectrum (MS-ESI) of compound **2j**

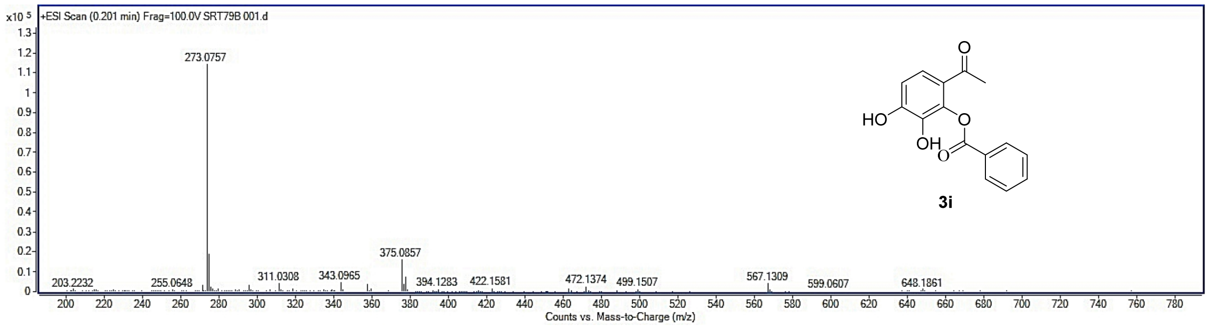

Figure S35 Mass spectrum (MS-ESI) of compound **3i**

.

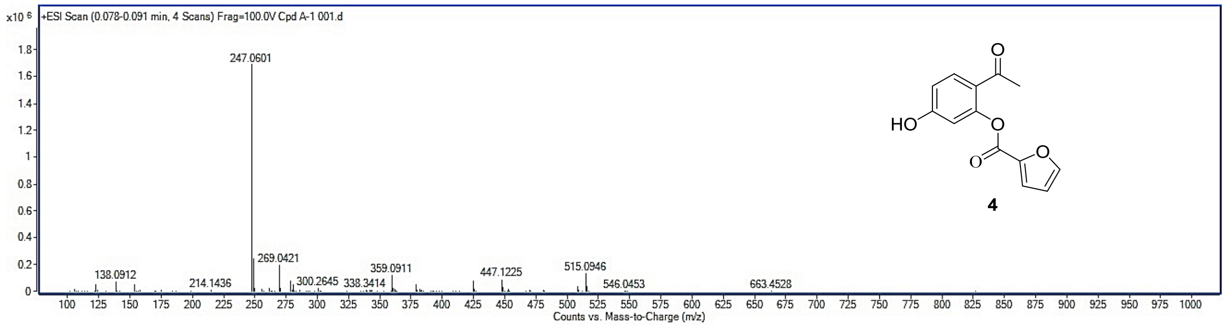

Figure S36 Mass spectrum (MS-ESI) of compound **4**
